# Observing formation and evolution of dislocation cells during plastic deformation

**DOI:** 10.1038/s41598-025-88262-3

**Published:** 2025-03-13

**Authors:** Albert Zelenika, Adam André William Cretton, Felix Frankus, Sina Borgi, Flemming B. Grumsen, Can Yildirim, Carsten Detlefs, Grethe Winther, Henning Friis Poulsen

**Affiliations:** 1 Department of Physics, Technical University of Denmark, Kongens Lyngby, Denmark; 2 European Synchrotron Radiation Facility, Grenoble, France; 3 Department of Civil and Mechanical Engineering, Technical University of Denmark, Kongens Lyngby, Denmark

**Keywords:** Mechanical properties, Metals and alloys, Imaging techniques, Microscopy, Condensed-matter physics, X-rays

## Abstract

During plastic deformation of metals and alloys, dislocations self-organise in cells, which subsequently continuously decrease in size. How and when these processes take place has remained elusive, because observations of the structural dynamics in the bulk have not been feasible. We here present X-ray diffraction microscopy sequences of the structural evolution during tensile deformation of a mm-sized aluminium (111) single crystal. The formation and subsequent development of 40,000 cells are visualised. The cells form in a stochastic, isotropic and uncorrelated manner already at 1% strain. We reveal that the cell size and dislocation density distributions are log-normal and bi-modal distributions, respectively, exhibiting scaling and maintaining a fixed volume ratio between cell interior and cell boundary. This insight leads to an interpretation of the formation and evolution steps in terms of universal stochastic multiplicative processes. This work will guide dislocation dynamics modelling, as it provides unique dynamic data and understanding.

## Introduction

Metals and alloys are typically polycrystalline aggregates. When deformed plastically, dislocations are introduced into the lattice of each grain^1,2^. For materials with medium to high stacking fault energies the primary mechanism for facilitating the shape change is dislocation slip. The interplay between the plastic flow and minimisation of elastic energy implies that the dislocations organize into boundaries. Moreover the micro-structure often evolves to comprise two types of boundaries, on a coarser scale Geometrically Necessary Boundaries, GNBs, reflecting systematic variations in the plastic flow, and on a finer scale Incidental Dislocation Boundaries, IDBs, thought to represent statistically trapped dislocations^3^. The IDBs separate nearly dislocation-free regions called cells. With increasing deformation, the flow stress increases, and the entire hierarchical structure shrinks in length scale. Empirically, structural properties such as cell size and mis-orientation distributions have been shown to exhibit scaling with the applied field for plastic strains larger than 5–10%^4–6^, when separating IDBs from GNBs.

However, despite extensive studies - motivated by the socioeconomic impact of metals-we are still unable to predict the type of micro-structure that forms as function of material and processing from first principles. While correlations have been established with the active slip systems^7–9^, the mechanisms underlying the cell formation and cell sub-division are not known. This prohibits realizing the vision of “materials science in the computer” in this field. At the root of this predicament are two issues. Firstly, the complexity and computational effort in handling the large sets of dislocations involved has been prohibitive. Discrete^10–12^ and Continuum^13^ Dislocation Dynamics models (DDD and CDD) can at best simulate representative volumes up to about 0.5 and 1% strain, respectively. For that reason until recently even basic patterning has been elusive^14^.

Secondly, it is challenging to visualise the micro-structural evolution experimentally in a representative way, as the multi length scale problem requires a combination of contrast to cells and local dislocation content over a large representative volume, *in situ* and within the bulk of a sample. Traditionally, dislocation structures are mapped by electron microscopy (EM)^7,15–19^. EM provides very detailed maps, but is inherently limited in terms of representative volume by the use of thin foils or micro-pillars. Hence, the multiscale dynamics may not represent bulk conditions. For bulk studies x-ray diffraction based imaging has emerged as a powerful tool. Multi-grain X-ray imaging modalities such as 3DXRD and DCT can provide comprehensive information on the *grain* level, well suited for interfacing with crystal plasticity models^20–24^. X-ray scanning nano-beam methods on the other hand have been demonstrated to visualise a few dislocations^25–27^. For quantitative measurements of dislocation densities line profile analysis of x-ray diffraction patterns prevails^28–31^, but results are averages over grains or over the illuminated volume, and typically only represent a fraction of the dislocations present.

To address this multiscale experimental problem we have established Dark Field X-ray Microscopy^32^, DFXM. With modalities similar to dark field TEM^33^, this method enables large field-of-view visualisations of both dislocations^34–37^ and cells^32,38^ within the bulk. We here apply the technique to a comprehensive study of the structural evolution within Aluminum during *in situ* tensile deformation from an applied strain of $\epsilon = 0$ to $\epsilon = 0.046$. The inspected volume comprises $\sim$ 40,000 dislocation cells, providing excellent statistics in this initial patterning range, where the structure is little known and the overlap with the strain regime available in simulations is most prominent.

**Fig. 1:**
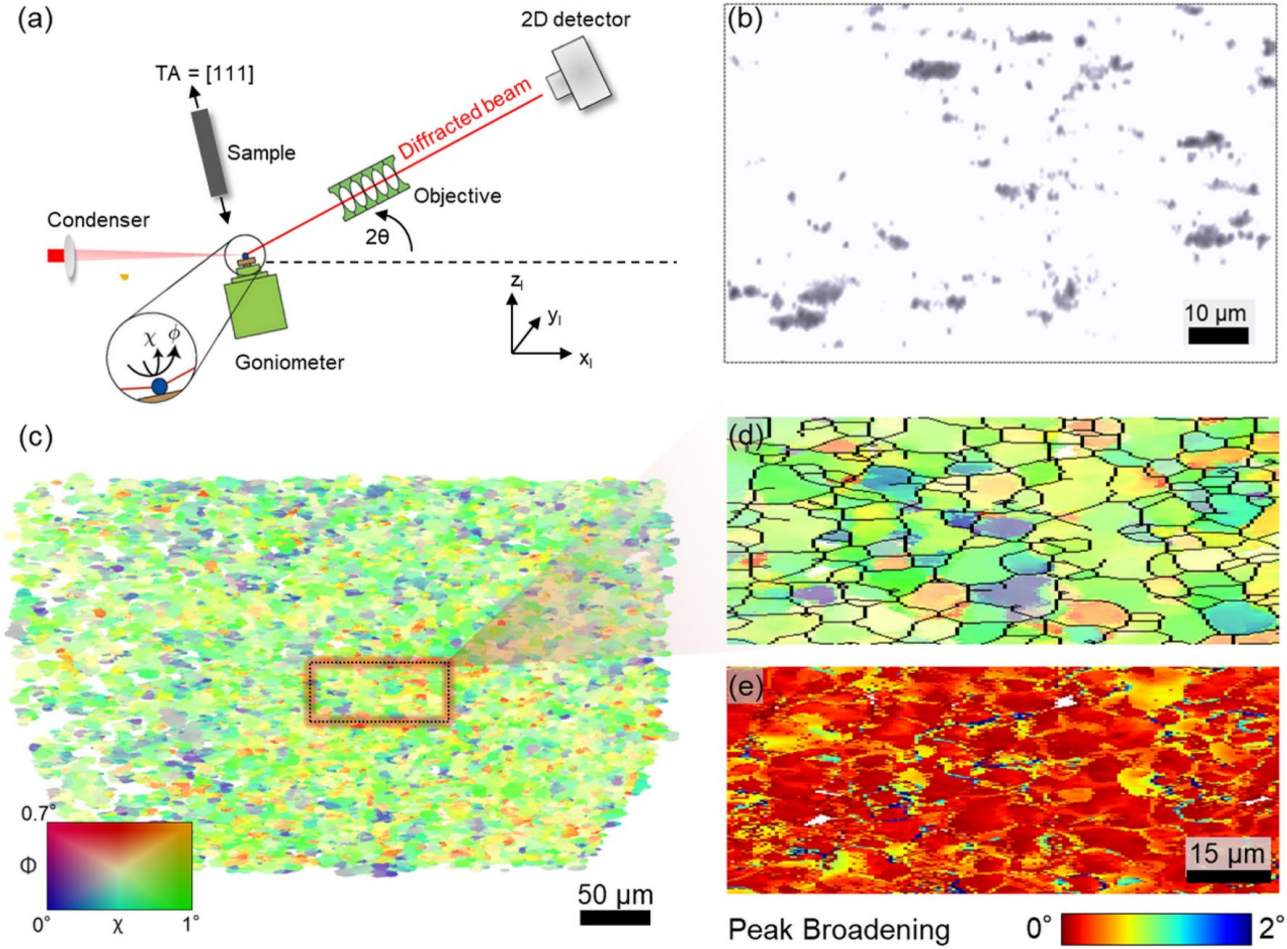
DFXM Geometry and examples of maps. (**a**) Sketch of DFXM set-up. The incoming x-ray beam is condensed in $z_l$ thereby illuminating one ($x_{l}, y_{l}$)-layer in the specimen. The objective provides a magnified image of the diffracted signal from this layer. The tensile axis (TA) of the sample is aligned with the diffraction vector. (**b**) Raw image in weak beam mode revealing dislocations and dislocation entanglements for applied strain $\epsilon =0.002$. (**c**) Orientation map for $\epsilon = 0.046$ with an Inverse Pole Figure color code. (**d**) Zoom-in on region marked in (**c**) with misorientations above 0.035° as black lines. (**e**) Corresponding map of the average peak broadening in directions $\phi$ and $\chi$, indicative of the total dislocation density.

The sample of choice is a single crystal of pure Aluminum, with [111] parallel to the tensile axis, see Fig. 1a. The mechanical device is mounted in the DFXM microscope such that diffraction in the vicinity of the [111] reflection is imaged for the illuminated layer. See Fig. S2 for more details of the loading device. By tilting goniometer angles $\phi$ and $\chi$, see Fig. 1a, contrast is provided to orientation and strain components. Three complementary modalities are illustrated in Fig. 1b–e. Weak beam contrast provides statistics over dilute dislocations ensembles at very low strains ($\epsilon \le 0.005$), orientation contrast provides statistics over cell properties, once these have formed, while the peak broadening $\Delta q$ is a proxy for the total dislocation density, $\rho$: $\Delta q \sim \sqrt{\rho }$. By repeating the mapping for a set of $z_l$ layers, a 3D map can be created. For definitions, algorithms and specifications, see sections 3.1 and 3.2.

Single crystals of the present orientation form parallel planar GNBs in addition to cells^39^. The GNBs are clearly manifest in both EM and DFXM when inspecting planes which include the TA (see Fig. S20 and Zelenika et al.^38^). In this work we report on the cell evolution primarily within a plane perpendicular to the TA. Here the GNBs are known to be less visible^39^. This was confirmed by the absence of preferred directions or orientation correlations between cells in the present data, see Figs. S11 and S12. This indicates that a model that does not explicitly take the crystallography of the deformation process (slip planes) into account may be adequate.

For $\epsilon < 0.01$, the sample exhibits a set of isolated dislocations and dislocation entanglements, as evidenced by Fig. 1b. In Section 3.6 these clusters are quantified in three complementary ways, which sample the elastic field associated with the dislocations differently. Consistently the three approaches reveal that the clusters are randomly positioned and distributed homogeneously within the Field-Of-View (FOV). Moreover their size distributions are consistent with log-normal distributions.

**Fig. 2:**
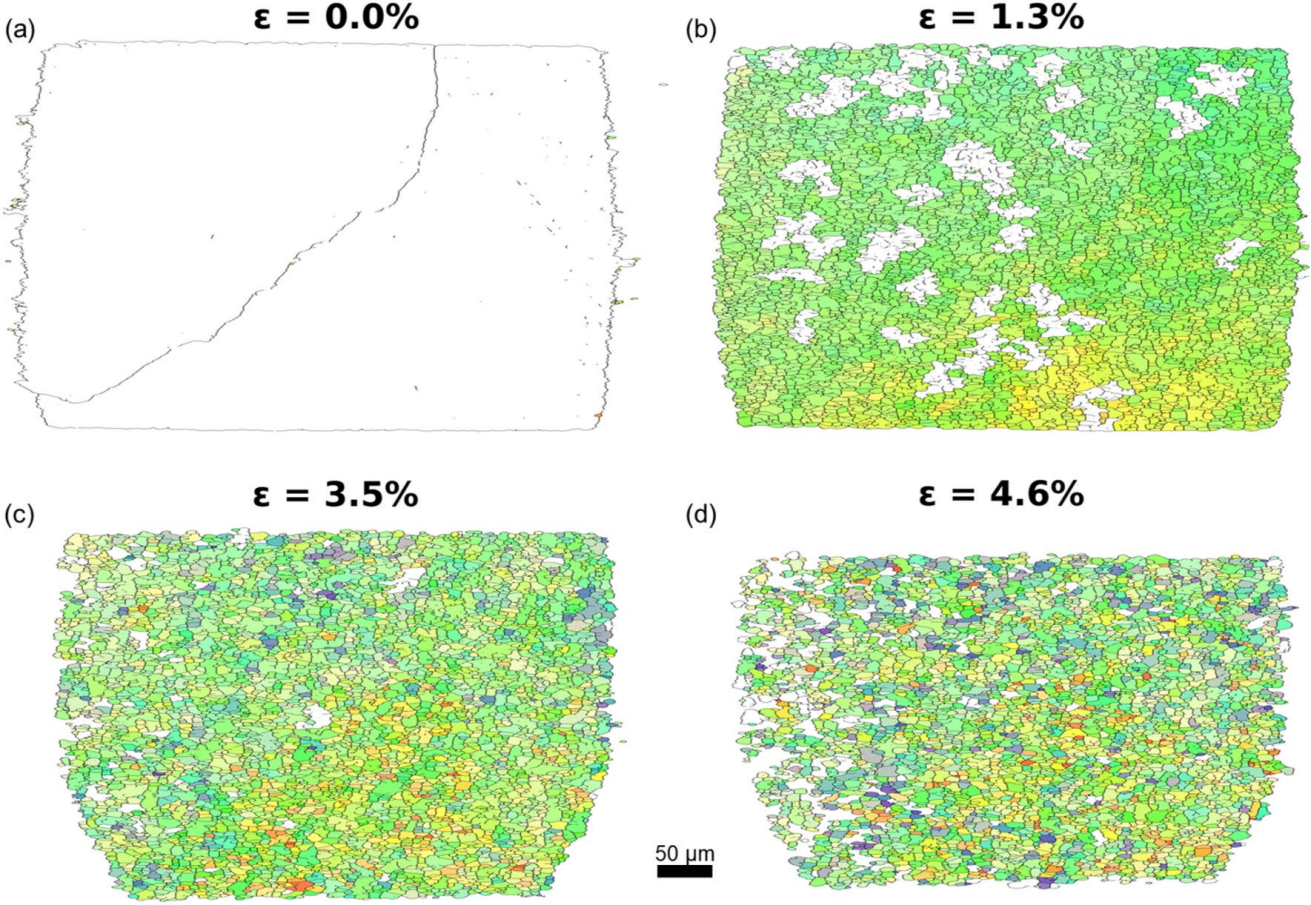
Cell formation. (**a**–**d**) Cells defined by a misorientation filter for selected applied strains, $\epsilon$, within the field-of-view of the microscope and for one of the 9 layers inspected. The color is indicative of the average orientation of each cell.

**Fig. 3:**
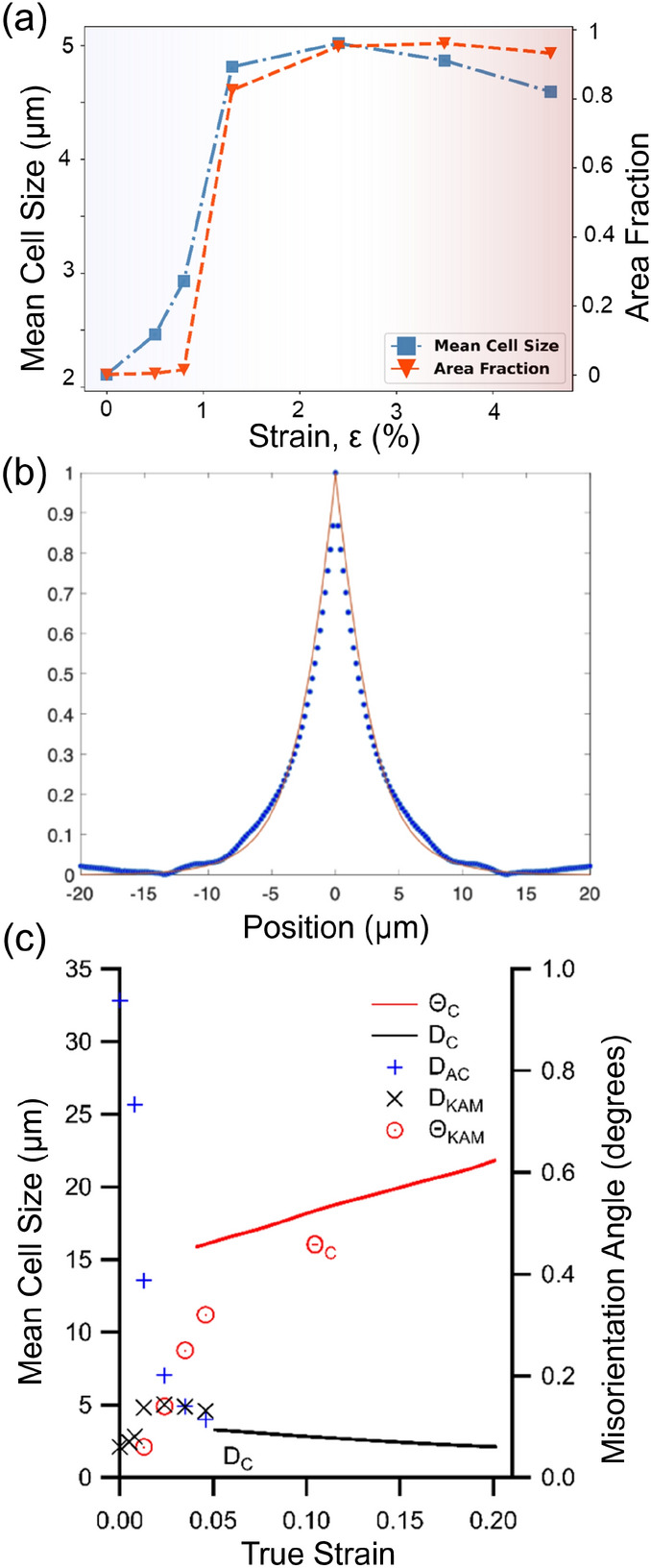
Structural order. (**a**) Area fraction of cells and average cell size as function of $\epsilon$. (**b**) Autocorrelation of the orientation map for $\epsilon = 0.046$ (blue points) along $y_l$ with a superposed model based on assuming the cells to be hard spheres (red line). All parameters in the model are provided by the experimentally determined cell size distribution. (**c**) Comparison of the properties measured by DFXM (symbols) with literature values based on TEM^3^ (lines). For the average cell size the literature values ($D_C$) are compared to the results of the cell analysis ($D_{KAM}$) and of the autocorrelation analysis ($D_{AC}$). For average misorientation, the literature values ($\Theta _C$) are compared to the cell analysis values ($\Theta _{KAM}$).

With increasing applied strain, the orientation spread and the peak broadening-both averaged over the entire volume inspected-grow approximately linear with the strain, see Fig. S5. Moreover, domains of approximately uniform intrinsic orientation distribution appear, see Supplementary Video 1 nd Fig. S21. We define cells as domains where the Kernel Average Misorientation, KAM, of all boundary voxels are above a threshold, $\theta _{\text {KAM}}$. By means of morphological operations, the boundaries are made 1 pixel thin, and as a consequence a tessellation of the sample is obtained, see Fig. S9. Anticipating scaling we set $\theta _{\text {KAM}}$ to be a linear function of $\epsilon$. The resulting KAM filter superposed on the orientation map are shown as function of $\epsilon$ as Supplementary Video 3 and Fig. S23. Snapshots are provided in Fig. 2. Inspection reveals that the tessellations are indeed similar, justifying the linear ansatz. This analysis also allows us to address a long-standing question: *when are the cells formed?* As shown in Fig. 3a the sample undergoes a transformation from no cells to site-saturation of cells in the range $\epsilon = 0.008$–0.023.

In a complementary approach we study the order in the structure on the micrometer length scale by deriving the autocorrelation function of the orientation maps. These are presented as function of $\epsilon$ in Supplementary Fig. S26. The absence of any side peaks clarifies that there is no long range ordering of cells. Next in Fig. 3b for $\epsilon = 0.046$, we compare the autocorrelation function with a hard sphere model of the cells with all parameters defined by the experimentally determined size distribution, see also Suppl. Information. The excellent match with experimental data informs that the cells “do not see each other”. We interpret this as evidence of the local and random nature of the cell formation process. From Fig. 3c it appear that this screening of the surrounding takes place as soon as the cells are fully formed, at $\epsilon = 0.024$. Moreover, it is shown that results are consistent with with the available TEM results for $\epsilon > 0.05$.

**Fig. 4:**
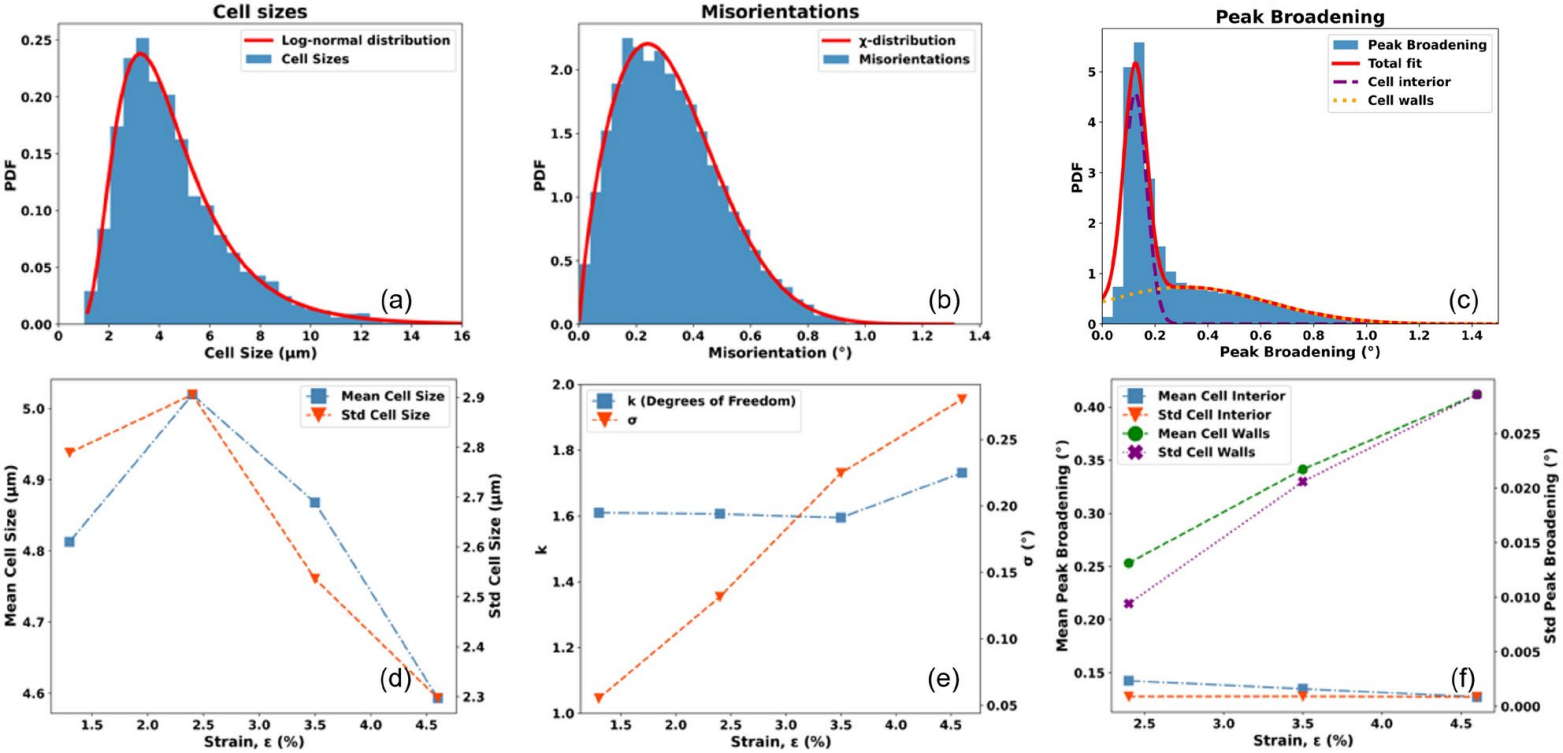
Statistical properties of the set of cells and their evolution with applied strain. Above: distributions for the 40,000 cells at $\epsilon =0.046$. (**a**) Cell size distribution with best fit to a log-normal distribution overlaid (red line). (**b**) Misorientation angle distribution with best fit to a $\chi$-distribution superposed (red line). (**c**) Distribution of the average peak broadening in $\phi$ and $\chi , \Delta q$, for each voxel. A best fit to two Gaussians is superposed (red line). The two components reflect predominantly elastic and plastic deformation, and are associated with the cell interiors and the cell walls. Below (**d**–**f**): corresponding evolution in the model parameters with applied strain. The results are consistent with scaling of all microstructural features with the external strain. In (**e**) k and $\sigma$ are the two parameters in the chi distribution.

The cell map at $\epsilon = 0.046$ comprises $\approx 40,000$ cells within the 9 layers analysed-one of them shown in Fig. 2d. This large ensemble allows us to distinguish with certainty between functional forms for various distributions, thereby strongly constraining models of structural evolution. The cell size distribution is found to be consistent with a log-normal distribution, see Fig 4a, while statistical tests reject other commonly used functions, cf. Fig. S13. Likewise, the distribution of misorientation angle between neighboring cells is well described as a $\chi$ function, Fig 4b. The corresponding peak broadening distribution, shown as Fig 4c, is a proxy for the local *total* strain. The distribution is bimodal, which we interpret as an elastic strain and a plastic strain component. The former is to a good approximation normal, within data uncertainty the latter may be normal or log-normal. As expected the plastic strain component is predominantly present in the rather broad cell walls, cf. Fig. 1e. Distributions of aspect ratio are provided in Supplementary Information.

Next, with the statistical tools presented, we describe the micro-structural evolution. Within the applied strain range from $\epsilon = 0.013$–0.046, where the material goes from predominantly formation (increasing cell sizes) to predominantly fragmentation (decreasing cell sizes), the distributions evolve in a consistent way, as summarised in Fig. 4d–f. Specifically, within statistical error the size distribution is log-normal throughout and exhibit scaling ($\sigma$ is constant.) Likewise, the misorientation angle distribution grows linear with strain and exhibits scaling (within experimental error k is constant). Moreover, the bimodal model for $\Delta q$ is valid throughout. The area fractions of cell interior and wall regions remain approximately constant, as do the profile of the elastic component associated with the cell interior. In contrast the wall distribution widens in a linear fashion with $\epsilon - \epsilon _0$, with $\epsilon _0 = 0.012$ being the onset of cell formation, cf. Fig. 4f.

The existence of a log normal cell size distribution throughout is consistent with *both* cell formation and division being multiplicative stochastic processes. This finding leads to suggest that the combined cell formation and division process can be described as a Markovian growth-fragmentation process^40^. Used e.g. in population science^41^ and chemical engineering^42^, such processes are mathematically proven to give rise to log-normal distributions and scaling. Specifically, our interpretation is as follows: individual dislocation entanglements appear randomly in time and space. Similar to particle creation by diffusion they grow with a growth rate that is proportional to their size. Following impingement, the cell pattern exhibits scaling with $\theta _{\textrm{KAM}} \sim \epsilon$. In the cross-over from formation to subdivision their area remains about constant, while new dislocations continue to build up the boundaries, the IDBs. The growth in dislocation density leads to a linear growth in the average misorientation across the IDBs. With the cells exhibiting no long range order, the IDB statistics can be modelled as a sum of stochastic processes, consistent with misorientations being associated with a chi distribution^43^. Finally, during fragmentation the larger cells are more likely to divide. This is corroborated by a positive correlation between cell heterogeneity and cell size, cf. Fig. S19.

In the past, log-normal-distributed plastic strain^44^, misorientation^45^ and geometrically necessary dislocation densities^46^ have been obtained experimentally with surface studies at the *grain* scale for polycrystalline metals deformed to strains exceeding $\epsilon =0.1$ and across a range of materials and operative micro-mechanical mechanisms like slip and twinning. Log-normal distributions have also been predicted for the plastic strain component by crystal plasticity simulations^47^ of *grain *ensembles, without any dislocations and for cell size and misorientation angle by assuming a cell splitting probability proportional to the cell surface area and misorientation evolution by rotational diffusion^48^.

Uniquely, in this work we study the deformation mechanisms at the length scale and through the strain range where the dislocation patterning (the cell formation) takes place. The DFXM maps shown here represent a representative volume deeply embedded in a sample sufficiently large that the mechanical conditions represents bulk conditions. The voxelated maps presented can be input to crystal plasticity FEM or CDD models. Subsequently two 3D movies may be compared: one experimental and one with the simulations. As demonstrated in the past e.g. for grain growth such a comparison of hundreds of thousands of points in space-time will provide unprecedented opportunities for further optimisation and validation of models^49^. For CDD such an approach also overcomes the limitation to the maximum strain range available in simulations today. The methodology presented applies to poly-crystals and all crystallographic space groups, limited mainly by the spatial resolution of 100 nm.

While this study is focused on the [111] tensile direction to study the formation of cells, other orientations with lower symmetry could exhibit a different cell morphology, as shown in previous studies^7,50^. Extending future research to include different systems would be valuable to gain further understanding on these differences.

To study the individual formation and fragmentation *events* we are commissioning a new goniometer and a more mechanically stable loading device, making it possible to track the micro-structural features in space and time by DFXM. Moreover, this may be complemented by mapping the local (purely elastic) longitudinal strain component, informing of the local stress state^51^. In our view the entire methodology established in this way-DFXM analysis and interfacing to modelling - is key for understanding a range of other processes in metals, such as annealing (recrystallization) and ductile damage, as it enables the combination of in situ mapping of variations in the deformed structure and local stress state with large volume identification of nuclei and voids, respectively.

## Methods

### Sample

The sample is a single crystal of 99.9999 % pure Aluminum of dimensions $1 \times 1 \times 20$ mm. The tensile axis is (111). After cutting, the sample was annealed at 540 °C for 10 hours.

The sample was mounted by glue on a grooved PEEK holder with a gauge length of 5 mm. This was inserted in a four-point bending loading device with the sample on the tensile side. In this geometry the sample is subject to uniaxial tension. The tensile device is illustrated in Fig. S2 and the resulting force-strain curve in Fig. S3.

### DFXM experiment

The DFXM experiments were conducted at Beamline ID06-HXM at the European Synchrotron Radiation Facility, ESRF. For details of the set-up see Kutsal et al. ^52^. A monochromatic beam with an energy of 17 keV was focused to a line with a FWHM of $\approx 600 \,\textrm{nm}$ in the $z_{\ell }$ direction, illuminating a layer within the sample. The scattering angle for the Al {111} Bragg reflection is $2\theta = 17.98$°. The objective was a Be Compound Refractive Lens (CRL), with 88 lenslets with radius of curvature $R = 50 \upmu$m, positioned at a sample-to-CRL-entry distance of $d_1 = 269$ mm and a CRL-exit-to-detector distance of $d_2 = 4987$ mm. The corresponding magnification and numerical aperture are $\mathcal {M} = 18.52$ (measured) and $NA = 0.705$ mrad (calculated), respectively. The 2D detector was located 5256 mm from the sample. With an additional magnification of 2 in the detector the effective pixel size was 656 nm (along $x_l$) $\times$ 202 nm (along $y_l$). The corresponding field of view in the sample is $350\,\mathrm {\upmu m} \times 900\,\mathrm {\upmu m}$. The scan parameters used are listed in Table 1 in Supplementary Information. The exposure time for a single image was 0.2 second including motor movements.

We did not observe any creep in the microstructure over the time of the acquisitions (minutes to hours).

### Data analysis

With the exception of the weak beam data, the entire analysis is based on the output of darfix^53^, as shown in Supplementary Videos 1 and 2. Specifically, for each voxel a 2D Gaussian is fitted to the $(\phi ,\chi )$ distribution. The subsequent analysis is detailed in Supplementary Information.

## Supplementary Information

Supplementary Material 1.

Supplementary Material 2.

Supplementary Material 3.

Supplementary Material 4.

Supplementary Material 5.

Supplementary Material 6.

Supplementary Material 7.

Supplementary Material 8.

## Data Availability

All the data presented in this paper, along with the analysis tools used for data evaluation, are available on GitHub at https://github.com/adcret/PMP. These resources include all data sets and scripts used to process and analyze the data following initial treatment steps with the darfix software. A comprehensive description of the analysis can be found in the cell_refinement_analysis.ipynb, further details about the packages and dependencies of this analysis tool are found in the README.md file. Data DOI: https://doi.org/10.15151/ESRF-ES-776857198
