# Supplementary material for: Observing formation and evolution of dislocation cells during plastic deformation
Source: Sci Rep. 2025 Mar 13;15:8655. doi: 10.1038/s41598-025-88262-3 (PMC11906882; doi:10.1038/s41598-025-88262-3)
Supplement: Supplementary file 1 — Supplementary Material 1. [file 41598_2025_88262_MOESM1_ESM.pdf]

Observing formation and evolution of dislocation cells during plastic deformation

A. Zelenika, A.A.W. Cretton, F. Frankus, S. Borgi, F.B Grumsen, C. Yildirim, C. Detlefs, G. Winther and H.F. Poulsen

Supplementary Notes I

3.1 DFXM methodology

The geometry of DFXM, coordinate systems, associated diffraction formalism and interfacing to micro-mechanical modelling is presented in detail in H.F. Poulsen *et al.*, J. Appl. Cryst. 50, 1441 (2017) and H.F. Poulsen *et al.* J. Appl. Cryst. 54, 1555–1571 (2021). while the details of the instrument at ID06-HXM, ESRF are presented in Kutsal *et al.*, 2019[52]. For convenience we summarise the definitions and optical properties relevant for this article.

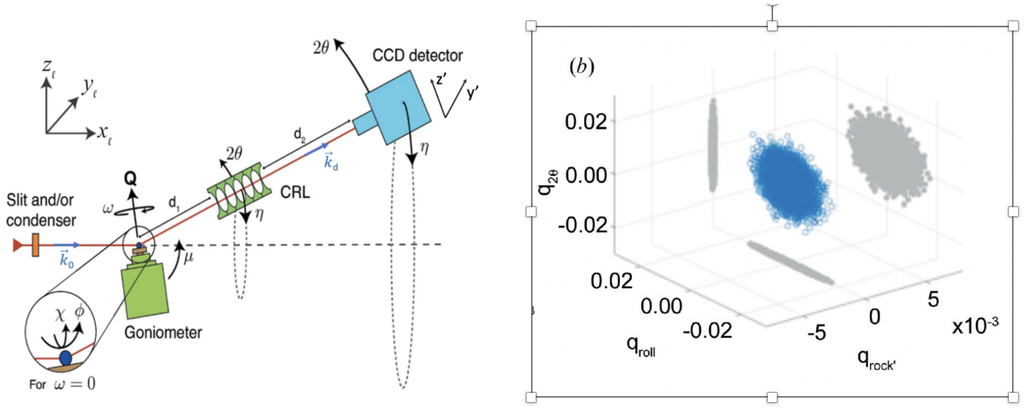

Figure S1. a) DFXM geometry as shown in the laboratory coordinate system, (x_ℓ, y_ℓ, z_ℓ). The optical axis before the sample corresponds to the average incident beam direction. The optical axis of the objective (a Compound Refractive Lens is co-linear with the average diffracted ray, when the sample is in the nominal position for the Bragg peak. The pivot point of the goniometer and sample is coincident with the intersection of the two optical axes. Vector $\vec{Q}$ defines the local scattering vector at a given point $\vec{r}(x,y,z)$ within the sample. The scattering angle is 2θ. The goniometer has four tilts, ϕ,χ, ω and μ. Only the former two is used in this work. b) Reciprocal space resolution function with units of strain, represented as a 3D scatter plot (blue). For ease of interpretation, the resolution function is projected onto the planes $\vec{q}_{rock} = 0, \vec{q}_{roll} = 0$, and $\vec{q}_{2\theta} = 0$ (all grey). This subfigure is adapted from H.F. Poulsen et al. J. Appl. Cryst. 54, 1555–1571 (2021).

The setup is illustrated in Fig. S1 a). A nearly monochromatic beam illuminates the sample. This beam is condensed in the vertical direction to generate essentially a line beam. The goniometer is designed to access diffraction angles in a vertical scattering geometry, and probe reciprocal space in the immediate vicinity of a given reflection, here $\vec{Q} = (1,1,1)$. The optical axis of the objective is aligned to the diffracted beam to produce an image on the 2D detector. The objective acts as a classical microscope magnifying an objective plane within the sample onto the detector plane. This microscope is characterised by the numerical aperture, NA, and the focal distance, f_N of the objective, as well as the magnification of the x-ray signal, $\mathscr{M}$, and the Field-of-View, FOV, in the sample plane. Due to the layered incoming beam, the DFXM images are affine transformations of the structure in the (x_ℓ, y_ℓ)-plane with an effective pixel size that is $1/\tan(2\theta)$ larger along x_ℓ than y_ℓ. To generate 3D maps the sample is translated relative to the incident beam in direction z_ℓ and acquisition is repeated.

In the simplified geometry used in this work, the goniometer has two angular degrees of freedom, the two orthogonal tilt directions ϕ and χ, with ϕ corresponding to rotation around y_ℓ. Diffraction contrast is obtained by scanning the sample in these two directions, known as rocking and rolling scans, respectively. These scans probe two shear components out of of the 9 components of the micro-mechanical tensor field, cf. H.F. Poulsen et al. J. Appl. Cryst. 50, 1441 (2017). As always, the two shear components comprise both a rotational and an elastic strain part. We cannot separate the two, but the magnitude of the field values implies that the rotation part is more dominant for the larger applied strains.

To probe the field of the axial strain, the "2θ-arm" - comprising both the objective and the detector - may be rotated corresponding to a shift of $\Delta 2\theta$. However, this modality was not applied in this work. Instead the scans performed are mosaicity scans, 2D grid scans in ϕ and χ, repeated for a number of layers. As a result each voxel in the illuminated part of the sample

are associated with a 2D distribution of intensity values, a function of ϕ and χ.

A 2D Gaussian model to this distribution provides in general good fits to this intensity distribution. As a result five parameters are derived for each voxel:

- *the Center-of-Mass, COM, in ϕ and χ.* We present these values in terms of poles in the (111) polefigure. Moreover, we quantify the evolution in the mosaicity map with ε in terms of texture evolution, by setting the third orientation axis, not measurable from only one diffraction vector, to a constant.
- *the normalised peak widths, Δq_ϕ and Δq_χ*, in units of strain. These are the widths (FWHM) of the fitted Gaussian in the two directions. To reduce complexity these results are combined by calculating the scalar width

$$\Delta q = \sqrt{\Delta q_\phi^2 + \Delta q_\chi^2}. \tag{1}$$

- *the amplitude*. In kinematic diffraction theory this is proportional to the fraction of the voxel that is illuminated. In practice this is also influenced by vignetting and by uneven sampling in reciprocal space caused by the objective.

Notably, the reciprocal space part of the experimental resolution function of the microscope is very asymmetric. A Monte Carlo simulation is provided in Fig. S1 b) representative of the setting used here. The width (FWHM) of the resolution function in direction χ is defined by the NA of the objective, here 0.705 mrad. In contrast the width of the resolution function in direction ϕ is dominated by the incoming divergence of the beam. This is 10 times smaller, of order 0.1 mrad. In comparison the local angular spread within the prestine sample was determined to be 2.0 mrad and 0.77 mrad, respectively. Moreover, the data acquisition algorithm involved a continuous scanning over ϕ where intensities are integrated over 0.04 deg = 0.69 mrad. Hence, it appears the finite angular resolution can be neglected for most of the work below.

3.2 Relationship between local peak broadening and dislocation density

The relation between the broadening of diffraction peaks and dislocation density is well studied when it comes to *the longitudinal direction*. Within the limitation of a dilute system the longitudinal strain can be described in terms of the dislocation density, ρ, as[31]

$$\Delta q_{long} \quad = \frac{\Delta d}{d} = \left[\frac{b^2}{4\pi} C_g \ln\left(\frac{R_e}{r_0}\right)\right]^{1/2} \sqrt{\rho}. \tag{2}$$

In this equation there are three constants: b, the modulus of the Burgers vector and R_e and r_0, the outer and inner cut-off of the dislocation system, respectively. The contrast factor C_g depends on the specifics of the active dislocation systems, but will be approximately constant during loading for the process studied here. This peak width purely reflects the elastic response.

In contrast broadening along the two *shear directions* will be combinations of elastic and plastic contributions, for higher applied strains dominated by the plastic spin part (rotation of the lattice). In Electron Back-Scatter Diffraction, EBSD, the misorientation between neighboring pixels is used to derive the geometrically necessary dislocation content, cf. W. Pantleon, Scr. Mater. 58, 994–997 (2008). The analysis can be seen as a generalisation of the simple relationship between the misorientation angle θ_{mis} across a dislocation wall comprising identical and equi-spaced edge dislocations with neighboring distance a and corresponding density ρ_{GND}. For small θ_{mis}

$$\theta_{mis}/2 = b/a \sim b\sqrt{\rho_{\mathrm{GND}}}. \tag{3}$$

The same procedure applies to DFXM, where it is possible to derive all tensorial components, provided a 3D map is acquired. However, the spatial resolution function has to be taken into account.

Thanks to its high angular resolution, DFXM offers another modality, based on the *local peak broadening in the shear directions* ϕ and χ. For each voxel a local (ϕ,χ) distribution is acquired. This informs of the total dislocation configuration. A full exposure is outside the scope of this article. Here we will rely only on the two second moments of the distribution, the normalised peak widths Δq_ϕ and Δq_χ introduced above. From these we define the scalar average normalised peak Δq by Eq. 1. By analogy to Eqs. 2 and 3 we make the ansatz that the measured Δq is proportional to $\sqrt{\rho}$ and that the proportionality constant is independent of ε within the range explored here.

It is at times of interest to determine a proxy for the dislocation density within a local region, e.g. in the vicinity of a cell boundary. The Gaussian approximation applied implies that

$$\Delta q^{\mathrm{region}} = \Big(\sum_{i\,\in\,\mathrm{region}} (\Delta q_i)^2 + KAM_i^2\Big)^{1/2}. \tag{4}$$

To provide absolute numbers for dislocation densities, it is required to use data acquisition schemes involving more than one scattering vector. However, we argue that the arguments and conclusions presented are not restricted by this limitation. In particular we note that for stochastic process, the central-limit-theorem - and its extension to multiplicative processes - implies that contributions from different dislocation families (probed by different scattering vectors) add in a way that conserves the shape of the distributions.

For more accurate data estimates of the dislocations density field the instrumental resolution function should be deconvoluted. In practice this neglect leads to a minor shift in the elastic peak in Fig. 4c and similar plots.

3.3 Load frame and stress-strain curve

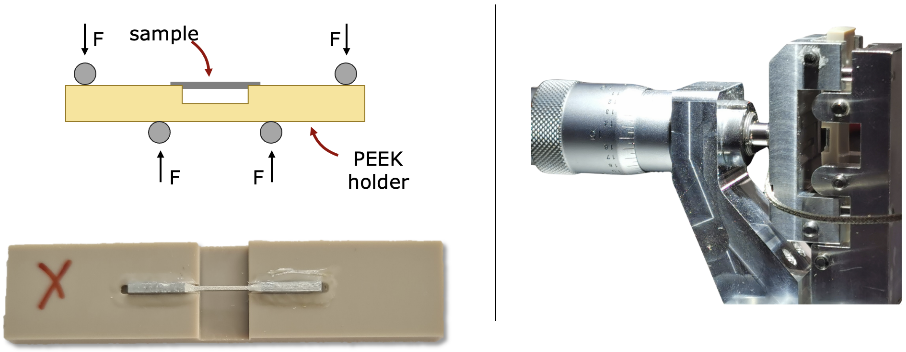

Figure S2. Principle of the tensile loading device. Left: sketch of four point bender illustrating the mount of the sample holder in a PEEK holder. Right: Realisation of the loading device.

The specimen is glued to the upper surface of a notched polymer holder that is subject to bending, see Fig. S2. Due to the small cross-section of the sample compared to the polymer holder and its position far away from the neutral axis, the load case is considered to be uniaxial tension. The bending is imposed through linear deflection of the two upper rolls of the setup using a micrometer screw. A "calibration" of the axial macroscopic strain in the sample and deflection in rig through the micrometer screw has been performed using an optical light microscope and DIC, see Fig. S3 (a). A "calibration" of the axial macroscopic strain in the sample and the deflection in the rig through the micrometer screw has been performed using an optical light microscope and DIC. Fig. S3 (a) measures the friction force applied by the screw, while Fig. S3 (b) shows a uniaxial tension experiment with a different sample geometry (cross-section of 1x1 mm^2). Strain in this experiment was measured with DIC and optical microscopy.

Between each DFXM scan the applied strain was increased manually, **and the position of the sample was centered by using the sample holder as a reference in the nearfield camera, with the probed volume remaining constant with an estimated uncertainty of $\sim$5 μm.** The strain rate is of order 10^{-4}/s. In Fig. S3 (a) we display the resulting relation between the force acted on the four point bender and the strain. Apparently, the force is nearly linear in the strain above the yield point.

3.4 Data acquisition scheme and overview of data analysis

Apart from weak beam data acquired for $\varepsilon \leq 0.002$ the scans performed during the *in situ* experiment are all mosaicity scans: two-dimensional scans of ϕ and χ. The scan parameters are listed in Table 1.

Due to small mis-alignments between load steps it is not the exact same set of layers that is mapped. Hence, tracking is not feasible with the mechanical device used, but statistical comparisons and tests are. To improve the statistics the analysis performed for $\varepsilon = 0.002, 0.005$ and 0.046 are based on 9 layers. (Tests proved the results for the individual layers to be identical within statistical error.) For reasons of computer resources, the analysis for the other strain step are based on the middle layer only. The experimental data for all layers and all strain steps are available in the metadata.

Most of the data analysis is based on the initial use of darfix for each layer. darfix generates pole-figures, cf. section 3.5 and (ϕ,χ)-distributions for each voxel. As described in section 3.1 the latter are generally well described as 2D Gaussian distributions. The resulting center-of-mass orientation maps and peak broadening maps are provided as Supplementary Videos 1 and 2, respectively. Note that for $\varepsilon = 0.035$ and 0.046 there are minor voids in the maps, caused by a lack of intensity, as these parts had orientations slightly outside the (ϕ,χ)-range mapped. Next cells are identified by the use of a Kernel-Averaged-Misorientation filter, see section 3.8,, Supplementary Video **4 and Fig. S24**.

This work is supplemented by an analysis of ordering on μm length scale, presented in section 3.7.

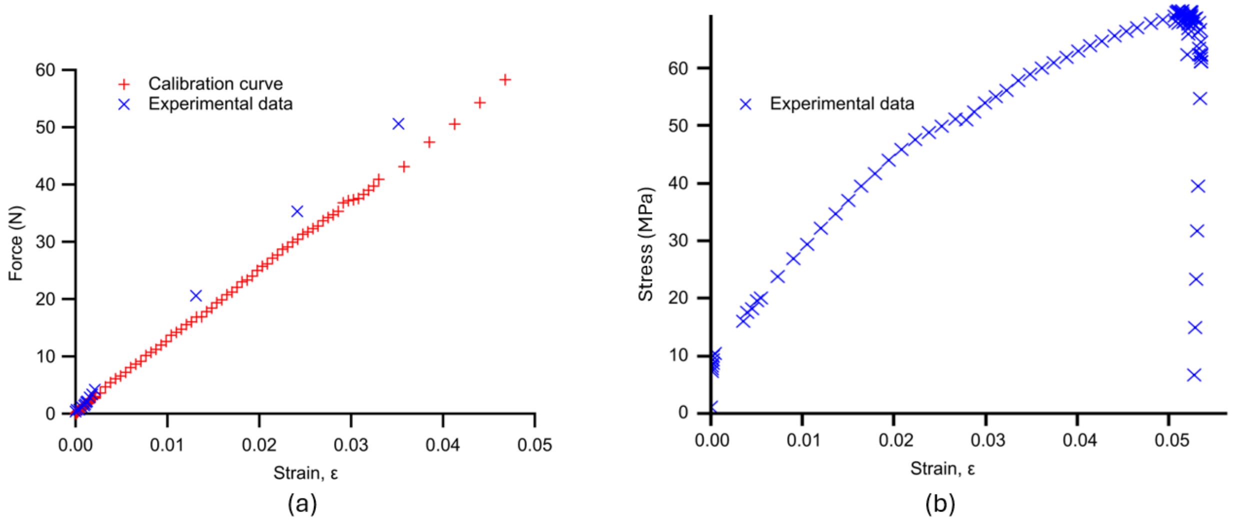

Figure S3. Force-strain **and stress-strain** curves for an aluminum single crystal. (a) Comparison between experimental data (blue crosses) and a calibration curve (red crosses) obtained from a similar sample using a 4-point bender loading device. This curve represents the friction force applied by the micrometer screw as a function of strain. (b) **Stress-Strain** curve for a similar sample deformed under uniaxial tension (cross-section of 1x1 mm^2), showing the typical stress response during deformation.

Applied strain	Nr mosalayers and optical magn.	Spacing in z	Scan ranges
0	1 in 2x and 16 in 10x	1 μm	0.6°in 30 steps in ϕ and 0.5°in 10 steps in χ
0.002	16 in 2x and 16 in 10x	3 μm for 2x and 1 μm for 10x	0.56°in 14 steps in ϕ and 0.56°in 14 steps in χ
0.005	16 in 10x	all 3 μm	0.56°in 14 steps in ϕ and 0.56°in 14 steps in χ
0.008	25 in 10x	9 with 5 μm , 5 with 3 μm, 11 with 10 μm	0.6°in 15 steps in ϕ and 0.6°in 15 steps in χ
0.013	11 in 10x	all 10 μm	0.68°in 17 steps in ϕ and 0.8°in 20 steps in χ
0.024	11 in 10x	all 10 μm	0.88°in 22 steps in ϕ and 0.96°in 24 steps in χ
0.035	11 in 10x	all 10 μm	0.88°in 22 steps in ϕ and 0.96°in 24 steps in χ
0.046	18 in 10x	3 with 5 μm, 11 with 10, 4 layers with 10 μm, interlaced with the 11 μm	0.88°in 22 steps in ϕ and 0.96°in 24 steps in χ

Table 1. List of mosaicity scans performed.

3.5 Macroscopic evolution

Initially we report on the evolution of structural properties when averaged over all voxels. Shown in Fig. S4 are the resulting *Pole figures* for the middle layer for strain steps $\varepsilon = 0, 0.013, 0.035$ and 0.046. It represents the summed intensity over all images. In the undeformed state the sample is a bi-crystal, with a mis-orientation between two domains of ~ 0.15 deg. At higher applied strain levels the distribution becomes an approximately isotropic Gaussian.

Shown in Fig. S5 a) is the evolution with ε in the resulting widths (FWHM) along ϕ and χ when fitting a Gaussian to the polefigures. Shown in Fig. S5 is the corresponding evolution of the local peak broadening. Apart from an offset at zero -

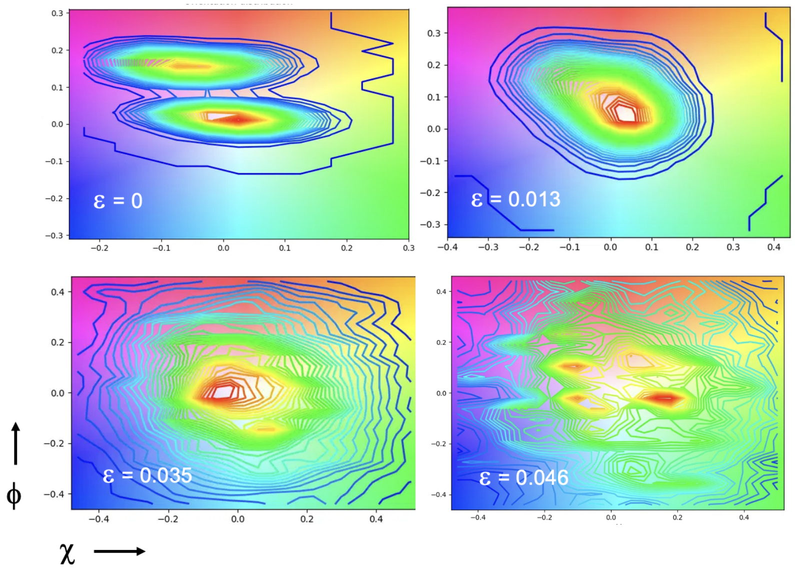

Figure S4. Pole figures for 4 applied strain levels with varying angular ranges.

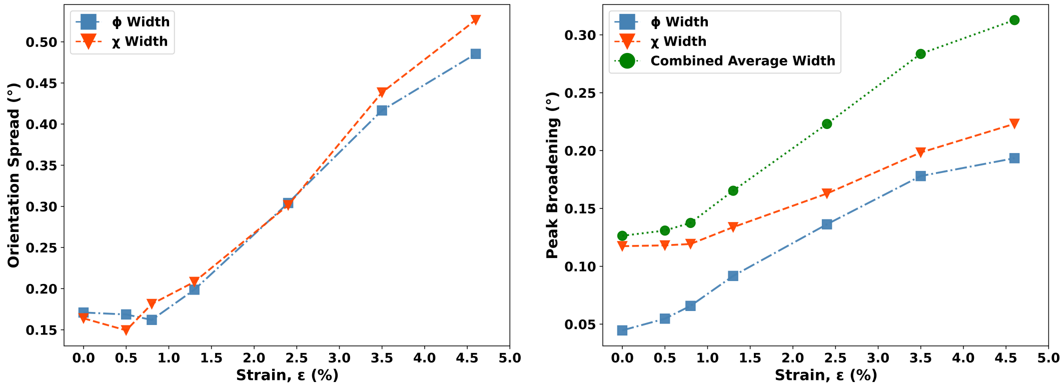

Figure S5. Macroscopic properties as function of applied strain. a) The width (FWHM) of the polefigure. b) Average values for the local peak broadening (FWHM).

reflecting that the pristine sample was not perfect - all parameters appear to be nearly linear in the applied strain. Moreover, we see that the local rotations (subfigure b) on average are approximately 1/3 of the global ones (subfigure a).

3.6 The pristine sample and the initial deformation

The pristine sample is a bi-crystal with a low-angle boundary exhibiting a mis-orientation of 0.15 deg, cf. the pole figure, Fig. S4, and the orientation map in Supplementary Video 1 **and Fig. S21**. This low angle boundary is clearly evident in the peak broadening map, cf. Fig. S6 a). The dislocation content appears approximately constant at a level of 0.32 deg, about twice the misorientation angle. We interpret the excess angle as indicative of an elastic strain in the boundary of ~ 0.005. The spatial width of the boundary is in some places defined by the resolution, at others it is extended by up to 1 μm, cf. Fig. S6 b).

Shown in Fig. S6 c) is the result of applying a threshold to the Δq map. A corresponding Kernel-Average-Misorientation (KAM) mask is shown in Fig. S6 d). In both cases, the resulting "dislocation clusters" appear isolated and approximately randomly distributed (with the exception of the low angle boundary). The individual clusters differs substantially in shape and size between in the two images: we attribute this to the difference in dislocation population and strain components and noise. In Fig. S6 e) and f) we have quantified the cluster size distributions using ImageJ. Given the populations size, both histograms are well described by log normal distributions.

The clusters as defined from thresholds on Δq or KAM are somewhat arbitrary, as they depend on the thresholds. As a complementary way to describe the dislocation ensemble, we can set a threshold on the weak beam image of the kind shown in

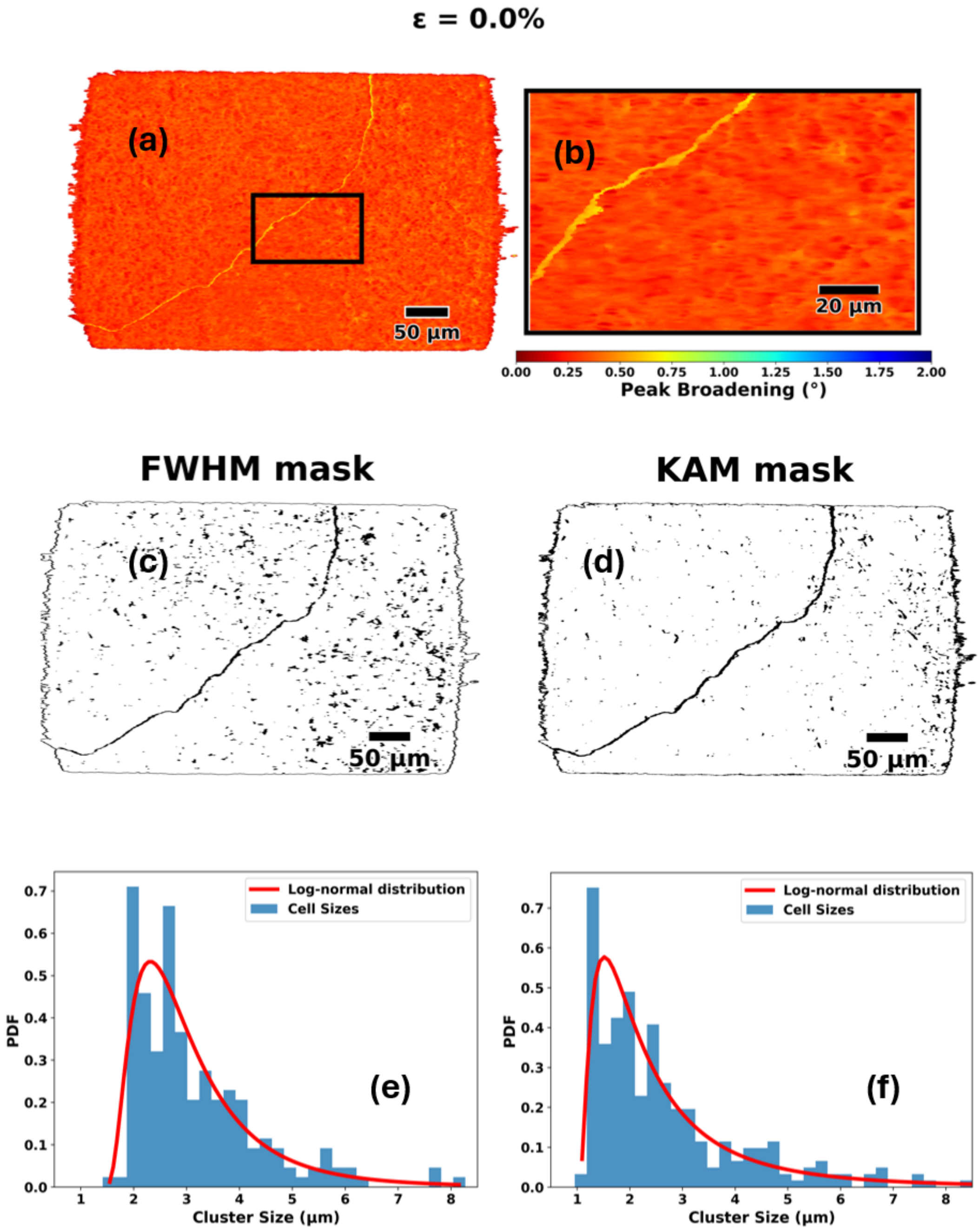

Figure S6. Properties of the pristine sample. a) the peak broadening map in false colors (the associated color bar is in degrees). b) Zoom in on region marked by a box in a). c) Segmentation of peak broadening map for full field-of-view, with a threshold of 0.195 deg. d) Corresponding KAM filter with a threshold of 0.01 deg. e) Histogram of cluster sizes in c) with a superposed best fit to a log-normal function. f) Histogram of cluster sizes in d) with a superposed best fit to a log-normal function.

Fig. 1 b). A histogram of the resulting size-distribution is again well described by a long-normal distribution.

Hence, three different analysis approaches all support the conclusion that the pristine sample comprise a set of dilute, non-interacting dislocation clusters, which are distributed randomly but homogeneously over the Field-of-View and with a size distribution which is consistent with a log-normal distribution.

3.7 Long range order: autocorrelation based analysis

In the context of images, autocorrelation measures the degree to which pixel values in an image are related to the values of their neighboring pixels across spatial intervals. Using an autocorrelation function to determine order is at the root of diffraction and crystallography. Following our previous work on an *ex situ* sample[38] we here use it **on Center-of-Mass (CoM) orientation maps** to determine order on the length scale of micrometers. The script for the 2D autocorrelation itself is the same as used in the previous work; essentially it is based on use of the MATLAB function xcorr2.

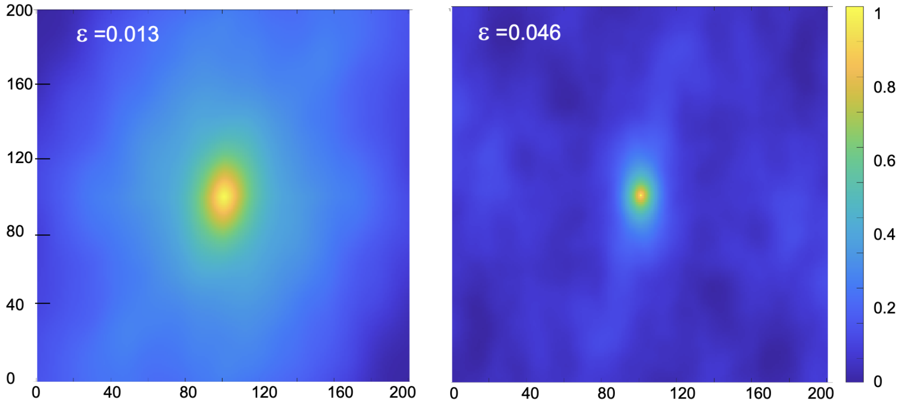

Figure S7. Auto correlation function for two applied strain levels

The autocorrelation function for the undeformed and the most deformed states are shown in Fig. S7. The projections along the center lines are shown for all applied strains steps in **Fig. S26**. The width (FWHM) of the central peak in the auto-correlation function along x_l and y_l are defined as the two "coherence lengths", $2\xi_x$ and $2\xi_y$, respectively. The resulting widths are tabulated in Table 2.

	0 %	0.8 %	1.3 %	2.4 %	3.5 %	4.6 %
$2\xi_x$	31.2	24	12.1	6.2	4.2	3.6
$2\xi_y$	10.2	9.1	6.1	3.3	2.5	1.7

Table 2. Widths (FWHM) of the 2D autocorrelation function in directions x_ℓ and y_ℓ: $2\xi_x$ and $2\xi_y$, respectively. They are listed in units of μm.

In contrast to the *ex situ* DFXM study on a similar sample but of a different orientation[38], there is here no evidence of long-range order emerging from the autocorrelation function. (The one exception are the side-loops along the y_l-direction for ε = 0 and 0.005 - these are due to the bicrystal nature of the pristine sample.)

The continuous decrease in the coherence lengths is attributed to the cell formation process. We note an anisotropy, with the coherent length in the x_ℓ-direction consistently being 2-3 times larger than the one in the y_ℓ-direction.

In Fig.S8 we present the autocorrelation function in directions x_ℓ and y_ℓ for $\varepsilon = 0.046$. Superposed are the autocorrelation of a hard sphere model with non-interacting ellipses with a size distribution given by the anisotropic log-normal distribution found in section 3.8. In order to compare the model and experimental data stereology has to be taken into account. The autocorrelation function measures cord lengths. As such the width, 2ξ is related to the area, A, by $2\xi = \frac{4}{\pi}r = \frac{4}{\pi\sqrt{\pi}}\sqrt{A}$. On the other hand, the size, s, determined from the KAM is defined as $s = \sqrt{A}$. Hence, the hard sphere model is scaled by a factor $\frac{4}{\pi\sqrt{\pi}}$. Given the fact that there are no free parameters in this comparison, this is an excellent correspondence, consistent with the model that cells are fully formed at these points in time. The autocorrelation profiles for $\varepsilon = 0.035$ and $\varepsilon = 0.024$ both exhibit a similar shape, but with decreasing applied strain the correlation lengths become larger and increasingly different from the cell size, consistent with the cell formation not being complete.

At $\varepsilon \leq 0.013$ the autocorrelation function appears to be a superposition of two functions, as shown in Supplementary **Fig. S26**. We interpret these data as evidence for a "two-phase" system. Part of the sample is in the process forming cells. Another part is still "undeformed" and this part consequently exhibits a longer correlation length.

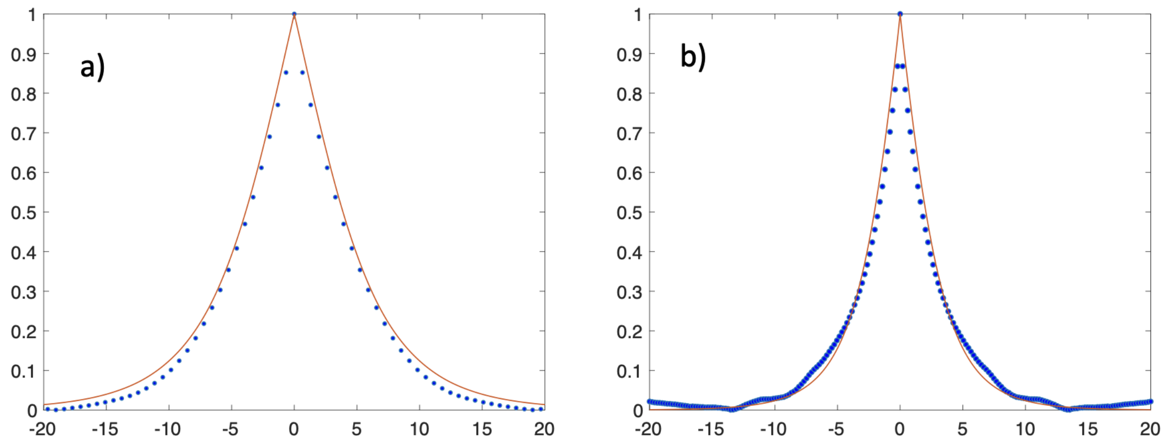

Figure S8. Comparison between experimental data for the autocorrelation (blue dots) and a hard sphere model with a log-normal size distribution for $\varepsilon = 0.046$. a) In direction x_ℓ, b) in direction y_ℓ. In both cases the x-axis is in μm.

3.8 Cell analysis based on a variable KAM threshold.

EBSD based analysis typically defines grains/cells by means of a mask derived from a map of the Kernel-average misorientation, KAM (see e.g. N. Lassen et al. Scanning Microsc. 6, 115–121 (1992) and K. Kunze. Textures Microstruct. 20, 41–54 (1993).) Following this tradition we will in the following define cells based on a threshold for the misorientation θ_{mis} and an isotropic kernel of size 2.

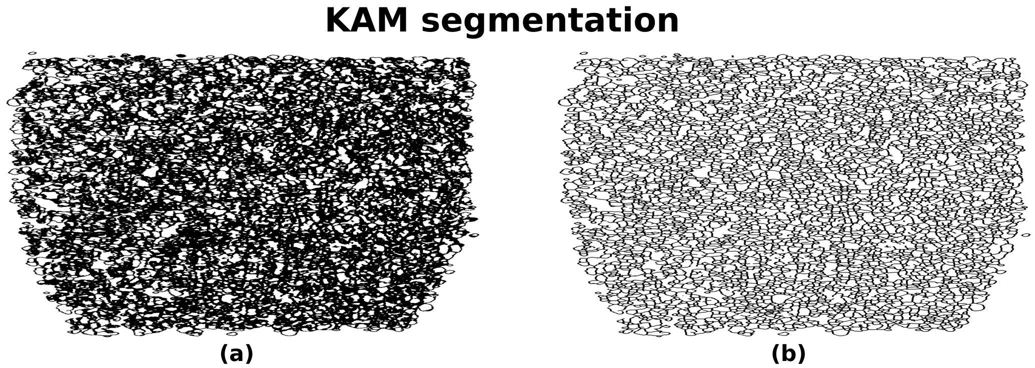

Figure S9. Example of KAM mask for $\varepsilon = 0.046$. a) Segmentation based on threshold of 0.034 deg in misorientation. b) corresponding KAM mask after morphological operations.

As we in DFXM with one reflection only map two orientation degrees of freedom, the definition for misorientation is revised to be

$$\theta_{\mathrm{mis}} = \sqrt{(\Delta\phi)^2 + (\Delta\chi)^2}. \tag{5}$$

Shown in Fig. S9 a) is a segmented image for $\varepsilon = 0.046$. The fraction of material below the threshold is in this case 71 %. Next morphological operations are applied to this binary image. Specifically, this image is skeletonized followed by a dilation of 1, implying that all cell boundaries have a thickness of 3 pixels. For the analysis in general the boundary thickness is 1 pixel. The resulting KAM mask is shown in Fig. S9 b). This is overlaid on the orientation map in the last frame in Supplementary Video **3 and Fig. S23**.

Based on an ansatz of a linear scaling with the applied strain we apply a variable KAM threshold that increases with the applied strain. Specifically, we keep the fraction of the segmented image above the threshold constant: $\theta_{\mathrm{mis}} =\sim 71\%$. This corresponds to thresholds of 0.10, 0.20, 0.32 and 0.41 deg for $\varepsilon = 0.013, 0.024, 0.035$ and 0.046 deg, respectively. The almost linear correlation is remarkable and justifies the linear ansatz.

The KAM algorithm leads to the generation of a site-filling map of domains. For lower degrees of applied strain one or more of these domains will represent the matrix: the undistorted part of the sample. Such domains (here defined by having areas larger than $(25\ \mu\mathrm{m})^2$) are removed from the set. So are domains with a size smaller than 10 pixel units. Finally, due to the (ϕ, χ) region scanned being a bit too small a minor fraction of the $\varepsilon = 0.035$ and 0.046 maps are filled with voids - again these regions have been removed. The remaining domains are identified as cells. The resulting cell maps are provided as Supplementary Video 1, with some of the frames also reproduced in Fig. 2.

The scipy function name enables the generation of statistics of the structural parameters embedded in the cell map. In addition, nearest neighbors are identified by dilating each cell slightly and detecting overlapping voxels. The volume fraction and mean cell sizes are reporoduced as function of the applied strain in Fig. 2. To enable a direct comparison between load steps, the number of cells and area fractions have been calibrated to the same total area. Other key statistical parameters are provided in Table 3.

ε	Number cells	Avg. size (in μm)	Avg. Anisotropy	Avg. misorientation (in °)
0.000	47	2.11	0.63	NaN
0.005	100	2.46	0.64	NaN
0.008	166	2.81	0.62	NaN
0.013	4033	4.81	0.59	0.06
0.024	4125	5.02	0.58	0.14
0.035	4383	4.87	0.601	0.25
0.046	4090	4.59	0.61	0.32

Table 3. Statistics of the cells generated by the KAM filter for a variable threshold of θ_{KAM}. The size is defined as the square-root of the area. The anisotropy is defined as the ratio between the major axis and the minor axis of the cells.

3.8.1 Cell shape anisotropy

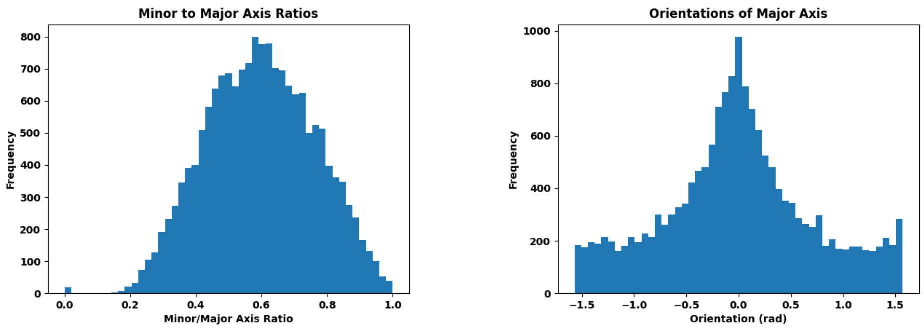

(a) (b)

Figure S10. Cell statistics on aspect ratios of the cells for $\varepsilon = 0.046$. The 0 orientation in b) corresponds to the direction of the x_l-axis.

Shape information arising from statistics on the $\varepsilon = 0.046$ set of cells is summarised in Fig.S10. The cells are on average elongated in the x_l-directions, consistent with the result of the auto-correlation analysis.

3.8.2 Cell mis-orientations

The local orientation relationships for the middle layer at applied strain $\varepsilon = 0.046$ have been characterised in two ways:

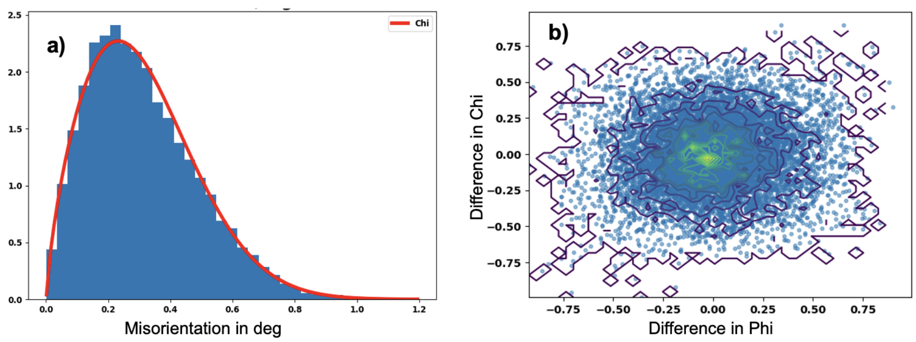

Figure S11. Orientation correlation between neighboring cells for $\varepsilon = 0.046$, I. a) Misorientation angle with best fit to a chi-function superposed. b) the misorientation axis. The contour line intensities are decreasing from (0,0) and outwards with a constant step in intensity.

Firstly, the misorientation angle and misorientation direction between neighboring cells is deduced from the set of cells generated above. The resulting mis-orientation angle and axis distributions are shown in Fig. S11. The sample appears to be isotropic to a high degree of accuracy.

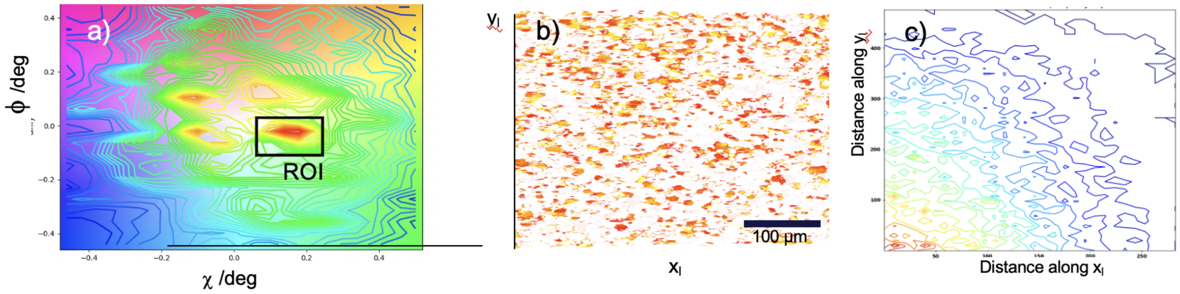

Figure S12. Orientation correlation for $\varepsilon = 0.046$, II. a) Polefigure composed of the COM positions with region-of-interest, ROI, superposed. b) Zoom in on cell map, showing only cells with an orientation within the ROI. c) A distance frequency map based on the set of cells identified in b). The contour line intensities are decreasing from (0,0) and outwards.

Secondly, as shown in Fig. S12 a) we define a region-of-interest, ROI, around one of the poles in the pole-figure. The area of the ROI is $0.1\,\mathrm{deg} \times 0.2\,\mathrm{deg}$. The subset of cells with orientations within the ROI, S_{ROI} are shown in Fig. S12 b). Next distances d_{ij} between center positions $((x_l)_i, (y_l)_i)$ of all cells $i, j \in S_{ROI}$ were calculated. The angular distribution of d_{ij} is shown in Fig. S12 c). Again within experimental error the data are completely isotropic.

3.8.3 Cell size distribution - comparison with theory

A survey of the compatibility of a set of model distribution functions with the cell size distribution generated by the KAM filter for $\varepsilon = 0.046$ is shown in Fig. S13. Visual inspection clearly identify the log-normal distribution as superior. A non-parametric test, the Kolmogorov-Smirnov, was used to quantify the fits. The p values listed in the legends in Fig. S13 represent the likelihood that the experimental data can be represented by the various distributions. With $p = 0.57$ the data are clearly consistent with the log-normal distribution, which is remarkable given the large population. On the other hand, with the usual threshold for statistical significance ($p_{\mathrm{limit}} = 0.05$) none of the other distributions pass the test. The fitting and tests were performed by standard Python SciPy code.

Remarkably, the log-normal distribution is a good approximation in the entire range where applying a KAM filter is meaningful: for $\varepsilon = 0.013$ to $\varepsilon = 0.046$, cf. Fig. S14. The resulting optimised values μ and σ are listed in Table 4 as well the mean (by definition equal to $\exp(\mu + \sigma^2/2)$. Also shown is the p value for the corresponding test.

3.8.4 Cells analysis with fixed KAM threshold

For completion we provide an analysis similar to that of section 3.8 for the case of keeping the KAM threshold fixed at the value optimised for $\varepsilon = 0.046$: $\theta_{\mathrm{mis}} = 0.034\,\mathrm{deg}$. Results for volume fraction of cells, mean size and cell size distribution are shown in Fig. S15 and S16, respectively. A summary of the log-normal cell size distribution parameters is presented in Table 5.

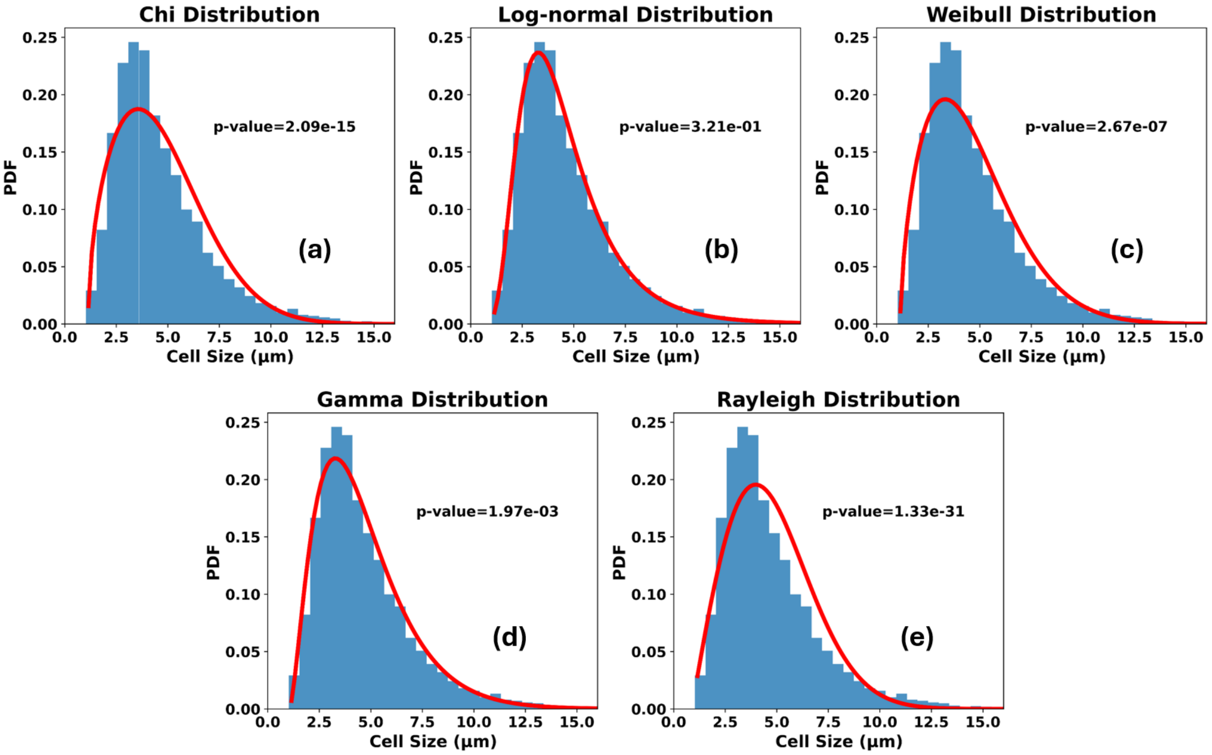

Figure S13. Test of models for cell size distribution for $\varepsilon = 0.046$. Superposed (red lines) are the best fits to model functions. Listed are also the results of Kolmogorov Smirnov tests.

ε	0.013	0.024	0.035	0.046
μ (in μm)	1.26	1.27	1.34	1.32
σ (in μm)	0.60	0.62	0.53	0.51
mean (in μm)	4.81	5.02	4.87	4.59
p	0.034	0.052	0.183	0.321

Table 4. Cell size distribution as defined by variable threshold θ_{mis}. Fitted values for the two parameters in the log-normal distribution: μ and σ. Also listed are the p-values from corresponding Kolmogorov Smirnov tests.

The conclusions in relation to the size distribution are identical to those obtained with a variable threshold, except for the cells forming later, as is to be expected.

ε	0.013	0.024	0.035	0.046
μ (in μm)	NaN	0.66	1.19	1.36
σ (in μm)	NaN	0.76	0.65	0.51
mean (in μm)	NaN	3.49	4.77	4.59
p	NaN	0.219	0.036	0.678

Table 5. Cell size distribution as defined by fixed threshold θ_{mis}. Fitted values for the two parameters in log-normal distribution: μ and σ. Also listed are the p values from corresponding Kolmogorov Smirnov tests.

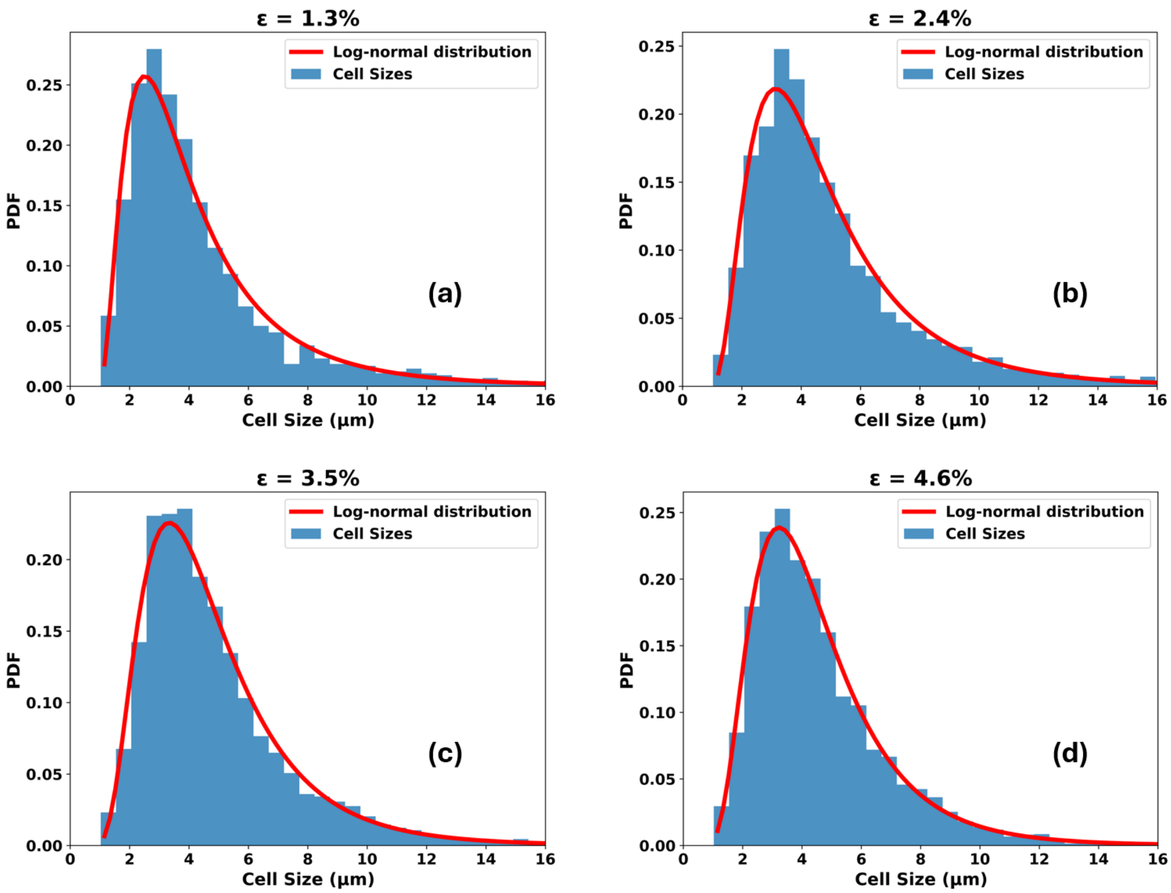

Figure S14. Log-normal fits to cell size distribution as function of applied strain, ε with variable misorientation threshold.

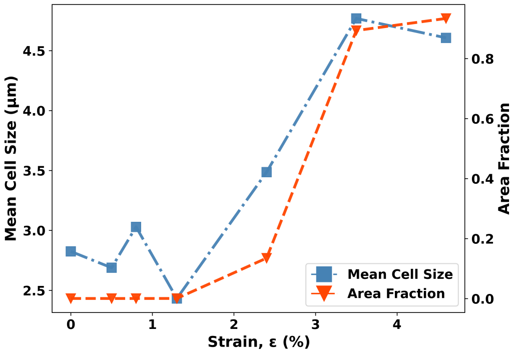

Figure S15. Area fraction of cells and average cell size as function of ε for a fixed misorientation threshold.

3.9 Dislocation density distribution

In Fig. S17 we plot the distribution of the average local peak broadening in directions ϕ and χ, as defined by Eq. 1. As described in Section 3.2 this is a proxy for the square root of the total dislocation density. It appears that a fit to a bimodal Gaussian distribution is satisfactory for $\varepsilon = 0.024$, 0.035 and 0.046. Following the modelling work by Chen et al.[47] it is natural to identify the two peaks as arising from regions where the response is elastic and plastic, respectively. We note that the statistics is not sufficient to determine whether the plastic component is normal or log-normal in nature. For convenience we have used a normal distribution throughout. Moreover, by inspection of the corresponding maps, see Supplementary video 2 **and Fig. S22**,

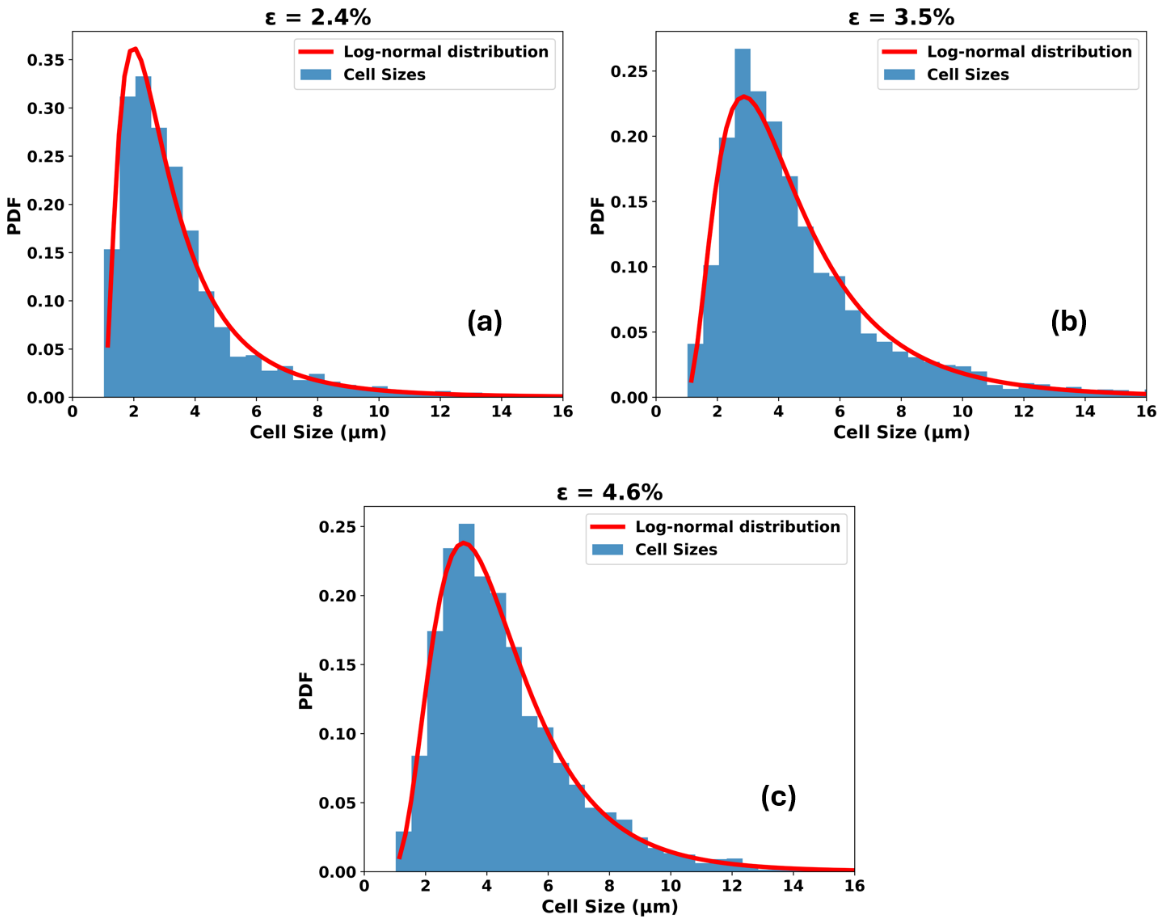

Figure S16. Cell size distributions as function of applied strain for a fixed misorientation threshold ε. Superposed are log-normal fits. For direct comparison with Fig. S14.

we infer that these two peaks approximately corresponds to nearly dislocation free regions and "stored dislocations regions": cell interiors and walls.

ε	0.024	0.035	0.046
Amplitude peak 1	3.30	3.58	3.43
Mean peak 1	0.19	0.18	0.17
sigma peak 1	0.04	0.04	0.04
Amplitude peak 2	1.40	0.67	0.51
Mean peak 2	0.35	0.45	0.45
sigma peak 2	0.10	0.19	0.29
Area ratio peak1/peak2	0.90	1.15	0.97

Table 6. Optimised parameters resulting from the bimodal Gaussian fits to the average peak width, cf. Fig. S17. The units of spatial variables is μm. Also shown is the ratio between the areas of the two peaks.

The resulting optimized parameters are provided in Table 6. To first order the area fraction of the two components is constant. As illustrated in Fig. 4 the average peak width of the dislocation rich component grows approximately linearly with the applied strain.

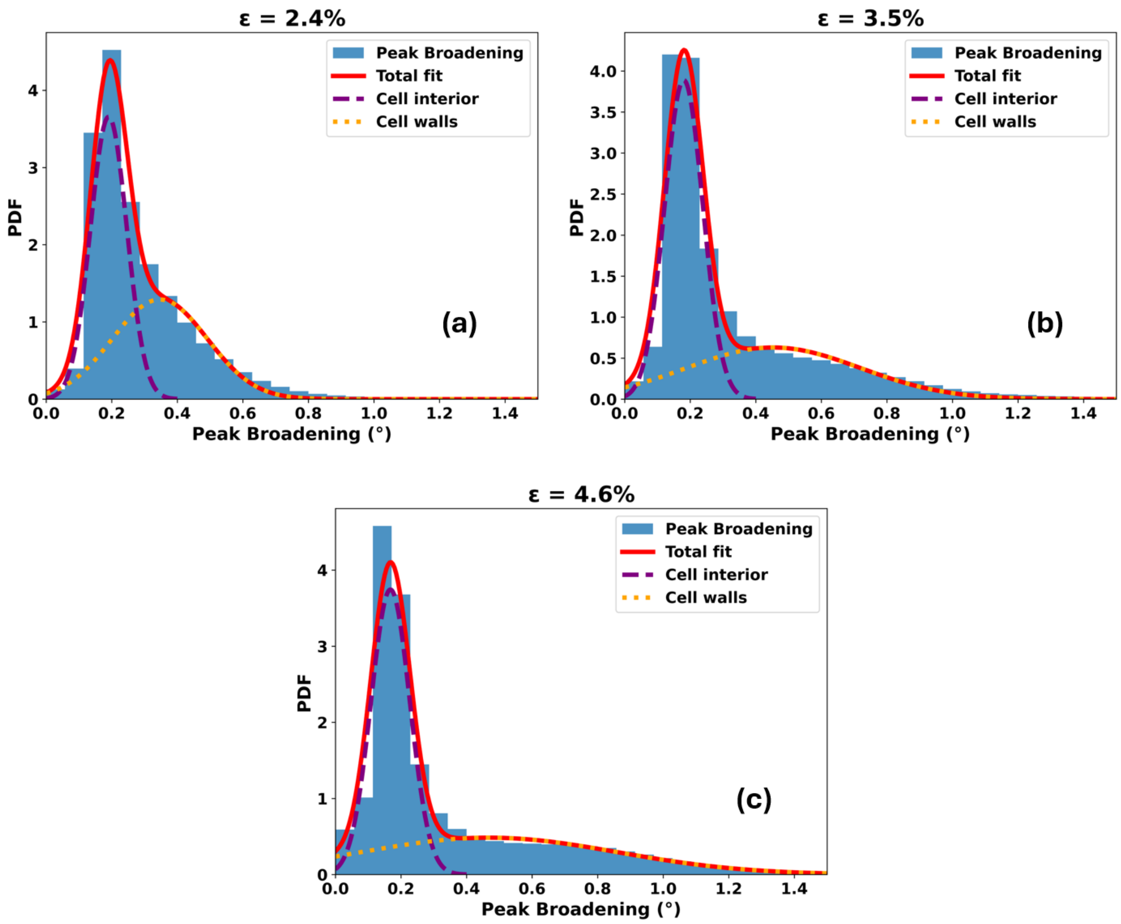

Figure S17. Distributions of peak broadening Δq as function of ε: a) 0.024, b) 0.035, c) 0.046. Superposed are best fits to a model comprising a bimodal Gaussian distribution (red curves). Also shown are the contributions to the model from the cell interiors (purple) and cell walls (yellow curves).

Shown in Fig. S18 is the spatial distribution of (the square root of) the total dislocation densities in relation to the cell boundaries, as defined by the KAM filter. Consistent with results from TEM the boundaries tend to decorate with dislocations within a boundary region of order 1 micrometer. in addition we identify some larger regions of relatively high density. Some cells have a high density throughout their area. Statistical tests shows that these are all relatively small cells. We attribute them as being the "bottom" or "top" of larger cells which have their center-of-mass outside the layer inspected.

Inspection also reveals that while most of the cells have a uniformly low density inside, some are heterogeneous in the sense that there are one or more subdomains with significantly larger density. Fig. S19 confirms the existence of a positive correlation between such heterogeneity and cell size. Speculating that such high-density sub-domains are precursors to new cells, and that the likelihood for fragmentation on average is linear in area, this insight outlines a mechanism that conforms with the concept of multiplicative stochastic processes. This *hypothesis* implies a birth process that is the opposite of the one in recrystallization, where the nuclei are less deformed than the matrix.

3.10 Comparison with literature values for larger applied strains

To the knowledge of the authors there are no quantitative microstructural data for the strain range studied in this work. In the following we compare with the work of Hansen[3] on TEM results of tensile strained polycrystalline 99.996 % pure aluminium in the range $\varepsilon = 0.05$ to $\varepsilon = 1.0$. Three characteristic types of microstructures appear as function of grain orientation. For [111]a the so-called type 3 structure was observed, a cell block structure where the dense dislocation walls are rotated by about 40 degrees to the TA. However, this characterisation was based on thin foil studies in a plane with a normal $\parallel$ TA. In contrast, TEM data acquired in the plane $\perp$ TA exhibited no discernible band structure[39]. The current data are acquired in a plane that is rotated by only $\theta = 10$ deg from the plane with a normal $\perp$ TA. The spatial anisotropy observed may be related to

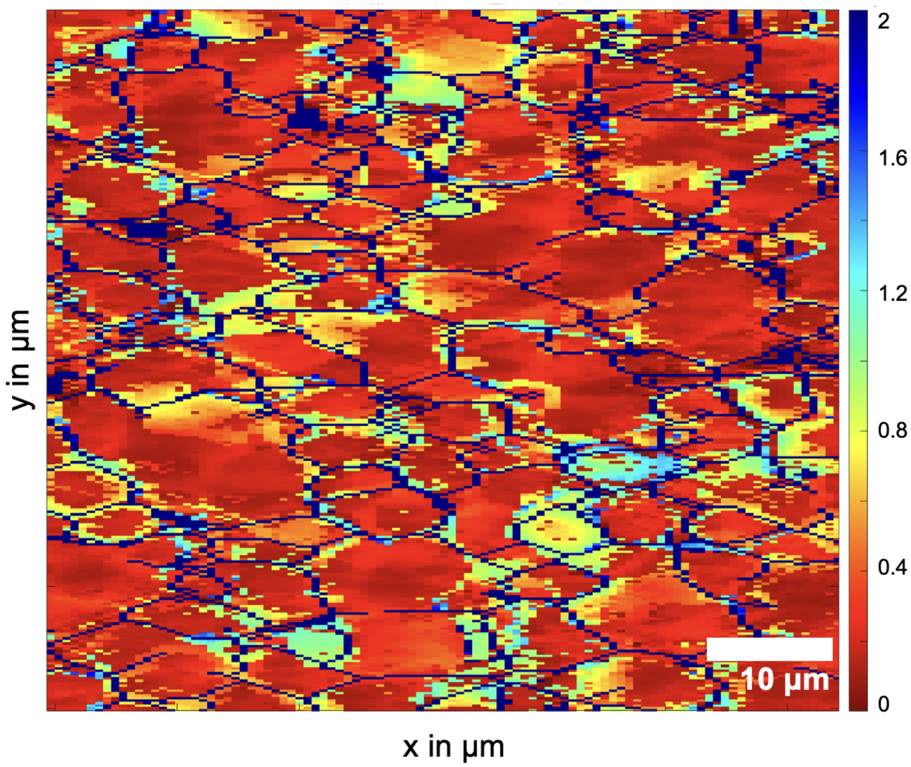

Figure S18. Region of interest of the peak broadening map for $\varepsilon = 0.046$ with angular values in degrees as presented by the color bar. Superposed is the KAM mask (dark blue lines).

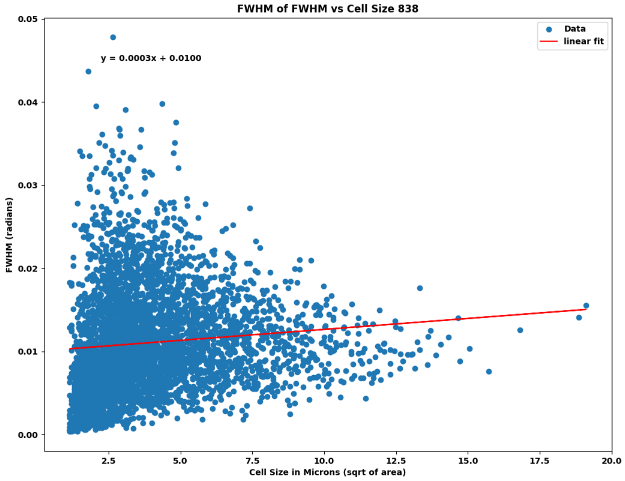

Figure S19. Correlation between cell sizes and the range of the peak broadening data, FWHM (Δq^{cell}), within each cell for $\varepsilon = 0.046$. Superposed is the best fit to a linear relationship (red line).

an out-of-plane ordering, but similar to TEM, otherwise there is no trace of band structure in the plane observed.

In Fig 3c we compare the average cells sizes and misorientation angles with the aforementioned TEM data. Given the different definitions of size, the TEM data should be multiplied by a stereological factor of $\pi\sqrt{\pi}/4 = 1.4$ for a direct comparison. In relation to the misorientation angle, on average the TEM values should be $\sqrt{3/2} = 1.22$ times larger, given

the fact that TEM measures all three components of the orientation while the DFXM data relates to only two. Given also the difference between single crystal and polycrystal samples, the correspondence with the TEM data is seen as excellent.

3.11 Complementary study on a (111) single crystal with the TA within the inspection plane

To confirm the finding from TEM that Geometrically Necessary Boundaries exist in this system, but are only visible when using a different inspection plane, cf. Section 3.10, we here report on a supplementary DFXM experiment on a different specimen, but with the same sample material and dimensions as in the main text. Moreover, the x-ray set-up was essentially identical, but performed after the microscope was relocated to the new dedicated DFXM beamline, ID03 at ESRF. Deformed to an applied load of $\varepsilon = 0.053$, the tensile axis was still [111], but the DFXM mapping was conducted using the (2-20) perpendicular to the tensile axis as diffraction vector.

The resulting orientation map is shown in Fig. S20. The orientation spread is similar but in this plane there is clear evidence of the GNBs.

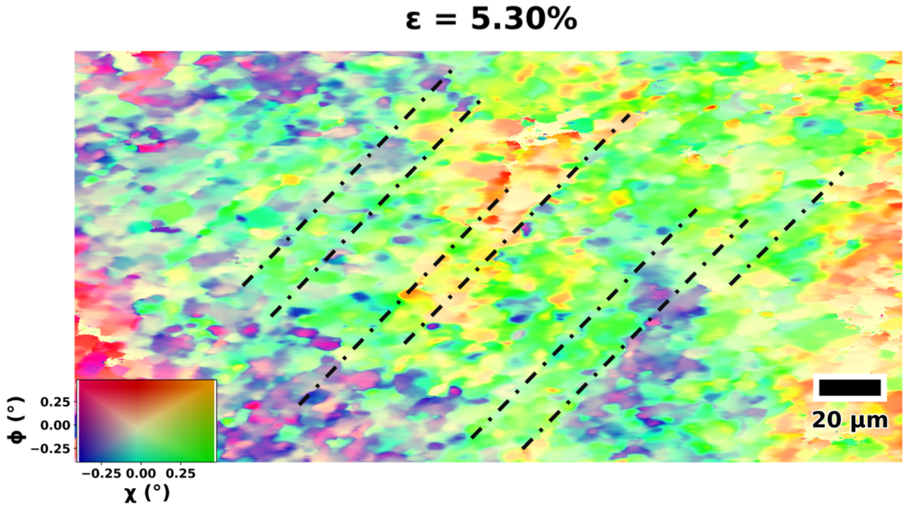

Figure S20. Orientation map for one inspection layer that is approximately orthogonal to the one in the main story for $\varepsilon = 0.053$. Overlaid, in black, are guides to the eye, identifying GNBs.

3.12 Mathematics in relation to log-normal and chi distributions

In this section we provide the parameterisations used in this work for the two distributions and we present the conditions for scaling.

The log-normal function,

$f(x)$ is a normalised function, parameterised as follows:

$$f(x) \quad = \frac{1}{\sqrt{2\pi}} \frac{1}{\sigma x} \exp\left(- \frac{(\ln x - \mu)^2}{2\sigma^2} \right) \tag{6}$$

With this parameterisation key properties of the distribution is given in Table. 7.

For $x \to \infty$ we have $f(x) \sim \frac{1}{x} \exp\left(- (\ln x)^2 \right)$.

To create a log-normal distribution with a given mean μ_X and variance $\sigma_X$we generate cells using the random function (where Z is a normal distribution)

$$X \quad = e^{\mu + \sigma Z} \text{ with } \mu = \ln\left(\frac{\mu_X^2}{\sqrt{\mu_X^2 + \sigma_X^2}}\right) \text{ and } \sigma^2 = \ln\left(1 + \frac{\sigma_X^2}{\mu_X^2}\right). \tag{7}$$

Mean	Median	Variance
$\exp(\mu+\sigma^2/2)$	$\exp(\mu)$	$\left[\exp(\sigma^2)-1\right]\exp(2\mu+\sigma^2)$

Table 7. Properties of log-normal distribution, with the parameterization expressed in Eq. 6.

Next, we establish the conditions for **scaling**. Consider two log-normal functions $f(x,\mu',\sigma')$ and $g(x,\mu,\sigma)$. Then these exhibit scaling with a factor k if and only if

$$f(x,\mu',\sigma') = kg(kx,\mu,\sigma). \tag{8}$$

For this to be true at all x we have

$$\mu' = \mu - \ln k; \qquad \sigma' = \sigma \tag{9}$$

The chi function
$f(x)$ is a normalised function:

$$f(x;\sigma,k) \quad = \frac{1}{2^{k/2-1}\Gamma(k/2)}\left(\frac{x}{\sigma}\right)^{k-1}\exp(-\tfrac{1}{2}(\tfrac{x}{\sigma})^2) \tag{10}$$

Here k represents the number of degrees of freedom. For $k=2$ it becomes the Rayliegh distribution, for $k=3$ the Maxwell-Boltzmann speed distribution. With this parameterisation key properties of the distribution is given in Table. 8.

	Mean, μ	Median	Variance
General $k>0$	$\sqrt{2}\frac{\Gamma((k+1)/2)}{\Gamma(k/2)}\sigma$		$k-\mu^2$
Rayleigh: $k=2$	$\sqrt{\pi/2}\,\sigma$	$\sqrt{2\ln 2}\,\sigma$	$(4-\pi)/2\,\sigma^2$

Table 8. Properties of chi distribution, with the parameterization expressed in Eq. 10.

To create a chi distribution with a given mean μ_X and degrees of freedom k we generate cells using the random function (where Z_i are normal distributions in the $i=1\dots k$ directions)

$$X \quad = \sqrt{\textstyle\sum_{i=1}^{k} Z_i^2}. \tag{11}$$

All chi distributions with same k scale automatically.

3.13 Data sequences

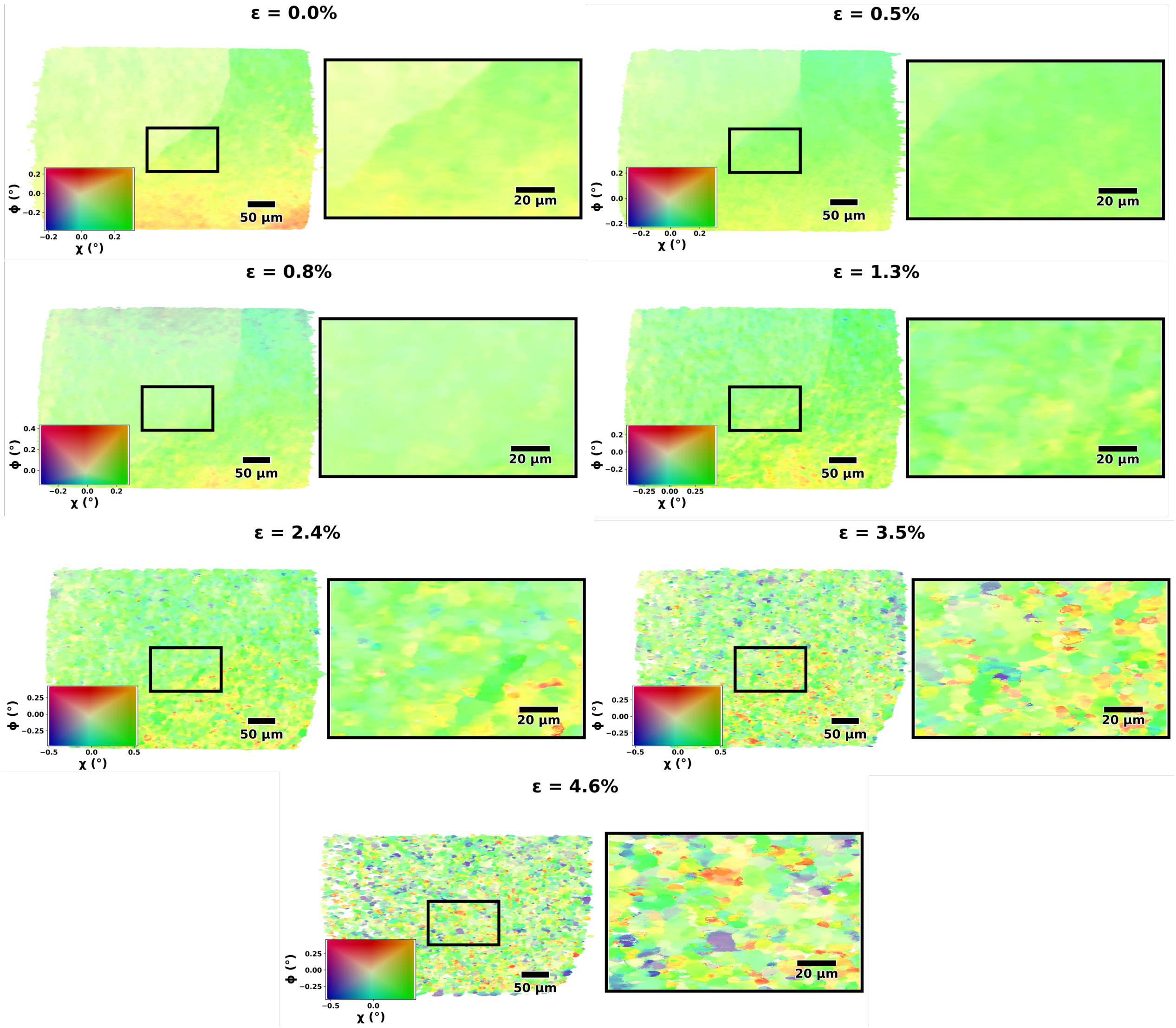

Figure S21. Data from supplementary video 1. Center-of-Mass (ϕ,χ) orientation maps. Left: the full field-of-view for the central layer with a region-of-interest, ROI, marked by the rectangular box. Right: a zoom-in corresponding to the ROI. The color scale employed varies from strain level to strain level and is indicated by inverse pole figures inserted.

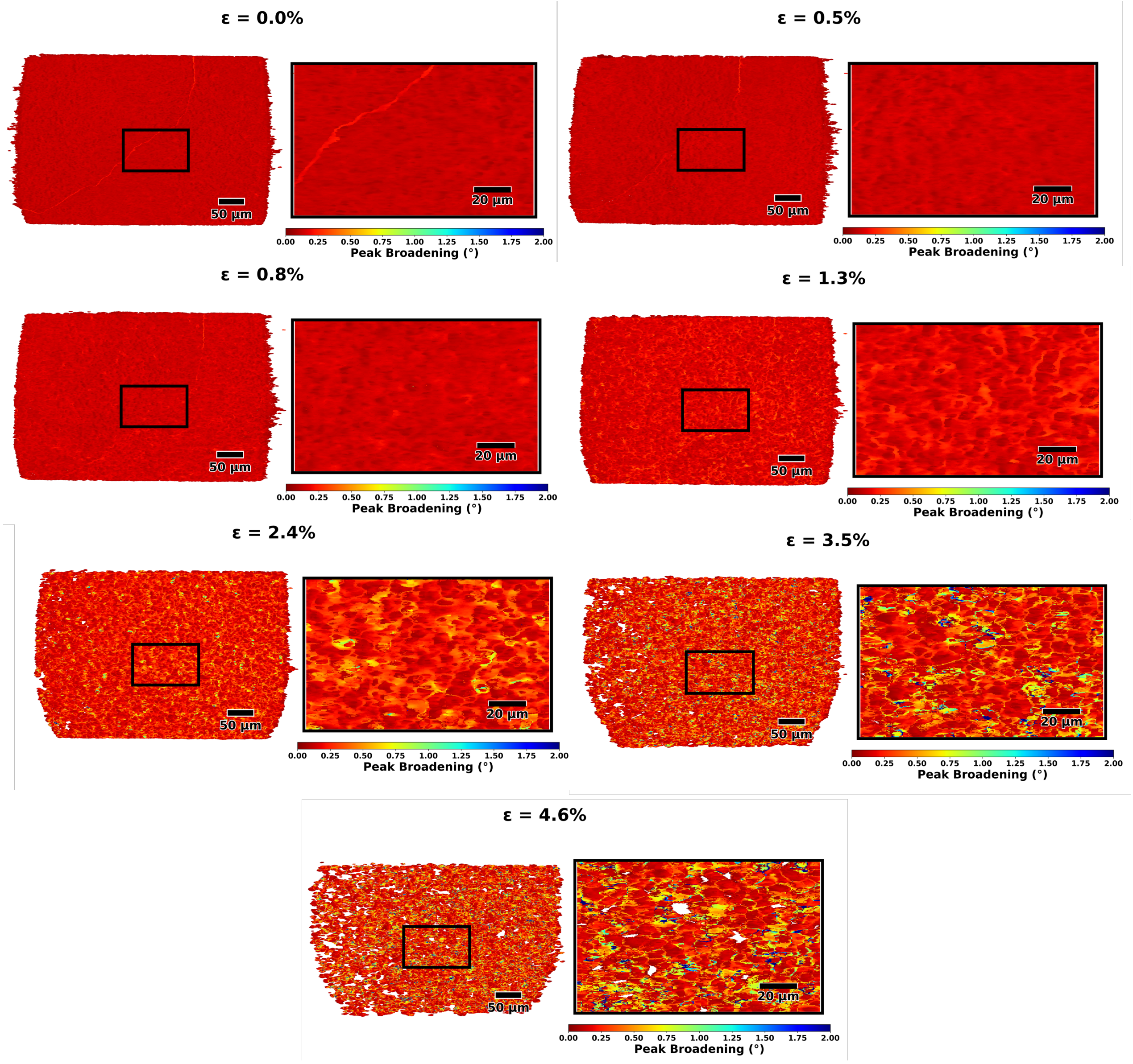

Figure S22. Data from supplementary video 2. The voxel-by-voxel width (FWHM) of the avearge peak broadening $\Delta q = \sqrt{\Delta q_{\phi}^2 + \Delta q_{\chi}^2}$, with Δq_{ϕ} and Δq_{χ} being the FWHM's resulting from a 2D Gaussian fit to the (ϕ, χ)-distribution. Left: the full field-of-view for the central layer with a region-of-interest, ROI, marked by the rectangular box. Right: a zoom-in corresponding to the ROI.

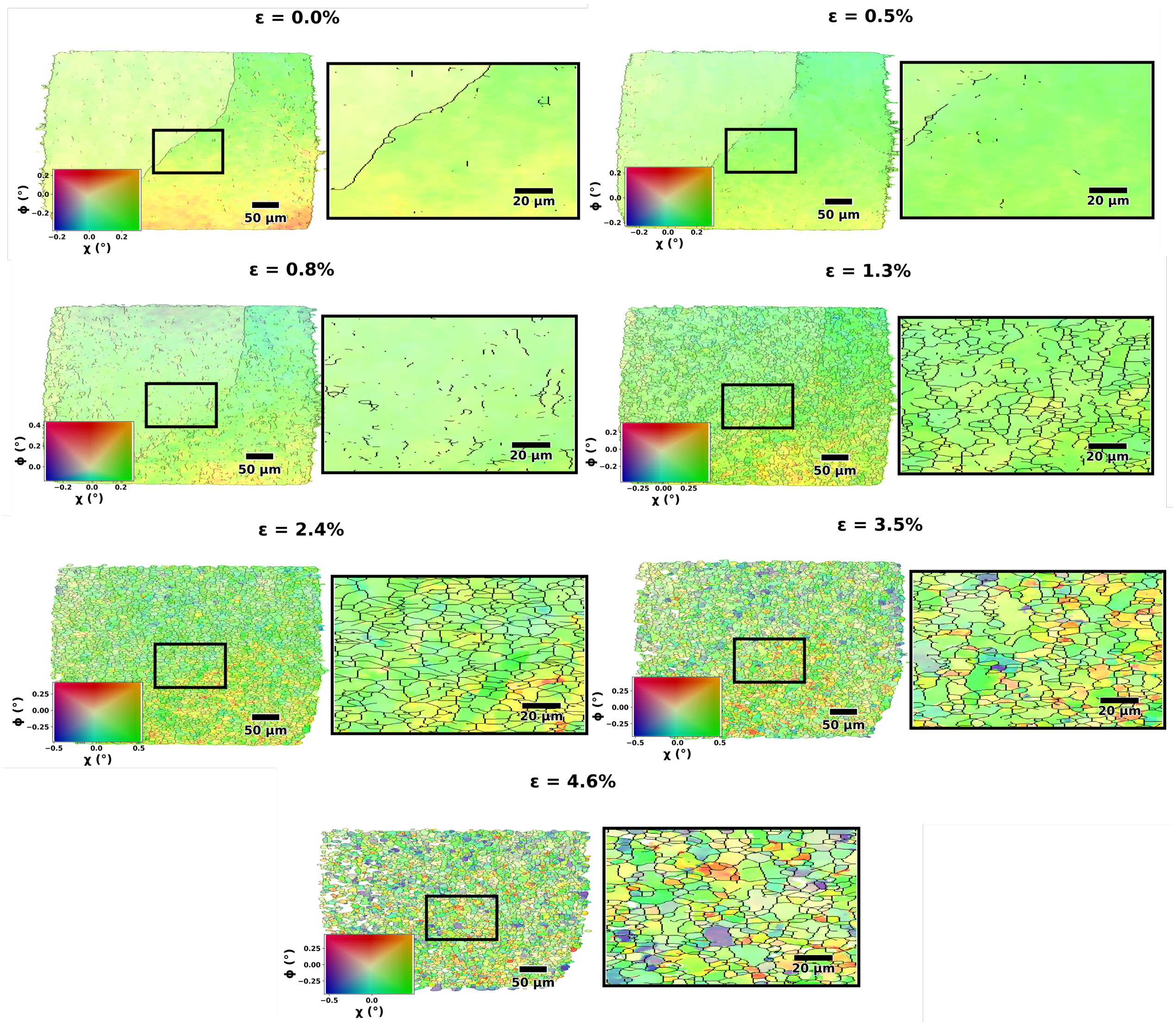

Figure S23. Data from supplementary video 3. Center-of-Mass (ϕ, χ) orientation maps with KAM mask overlay. Left: the full field-of-view for the central layer with a region-of-interest, ROI, marked by the rectangular box. Right: a zoom-in corresponding to the ROI. The color scale employed varies from strain level to strain level and is indicated by inverse pole figures inserted.

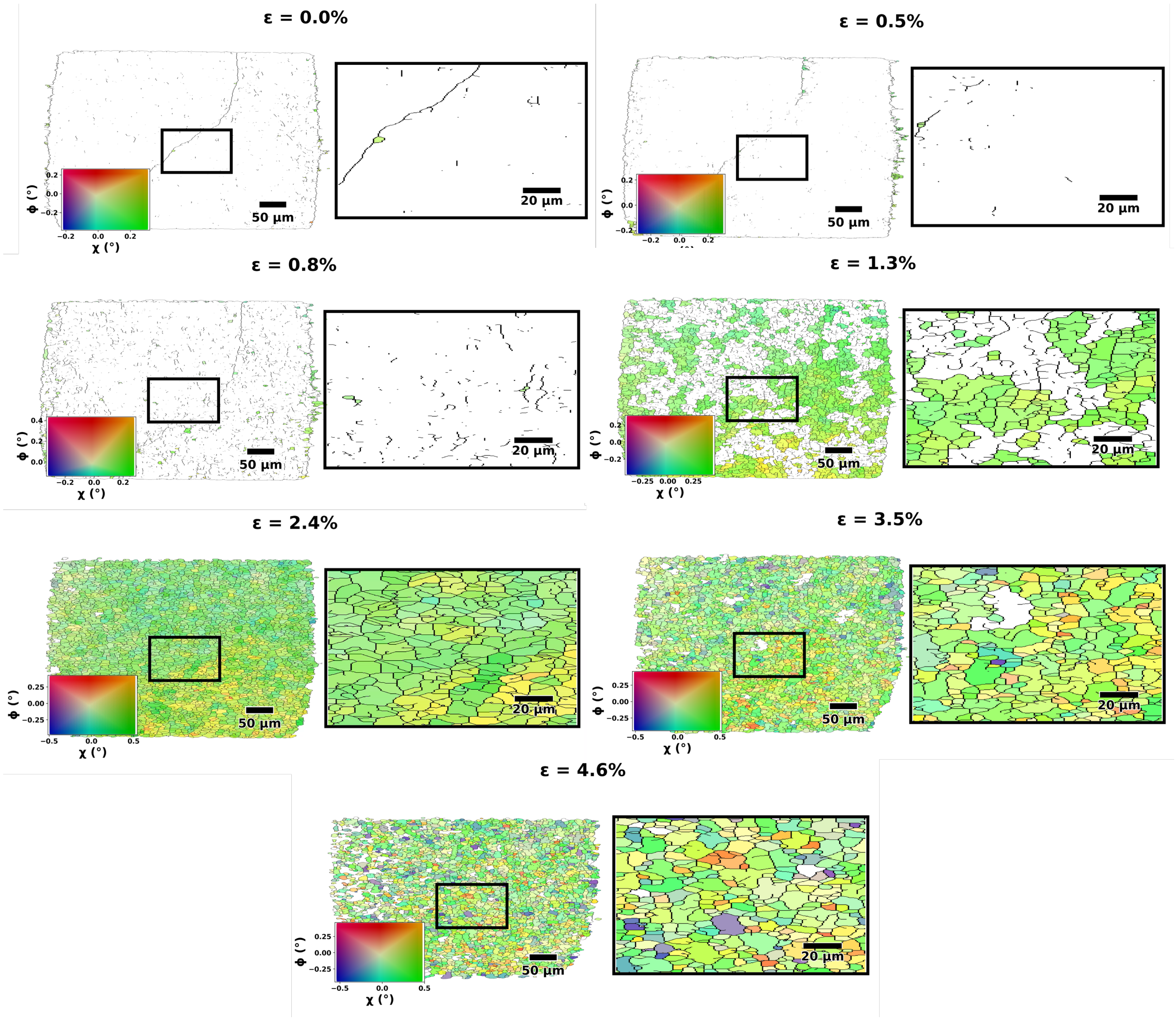

Figure S24. Data from supplementary video 4. Cells (colored regions) are identified as connected regions using a Kernel-Averaged-Misorientation (KAM) mask (black lines). The colors of the cells represent their average orientation, as indicated by the inverse pole figures inserted. Left: the full field-of-view for the central layer with a region-of-interest, ROI, marked by the rectangular box. Right: a zoom-in corresponding to the ROI.

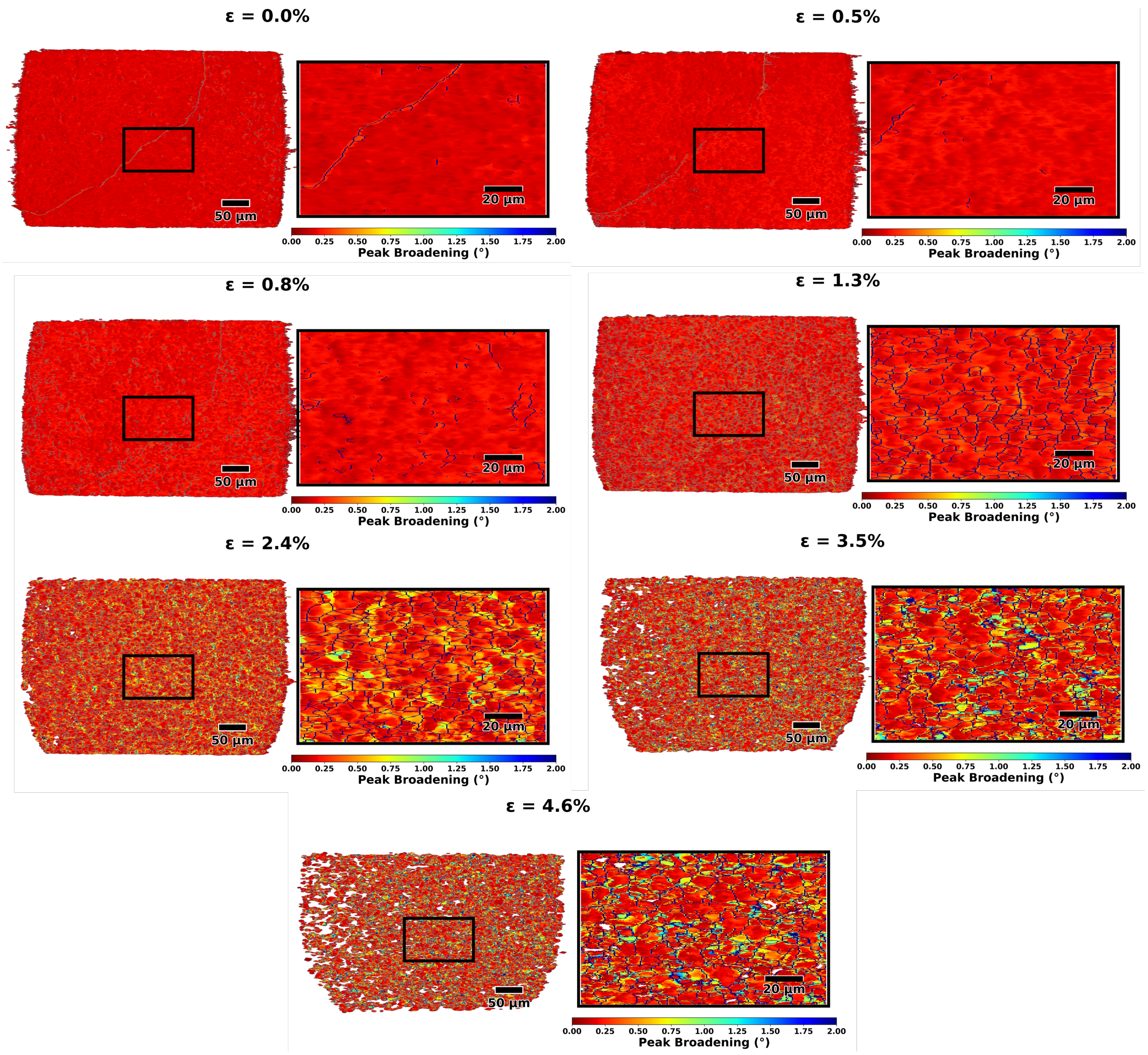

Figure S25. Data from supplementary video 5. Peak broadening maps with KAM masks overlaid. The images are replica of those in Supplementary Video 2, Fig. S22 with the KAM mask (black lines) overlaid.

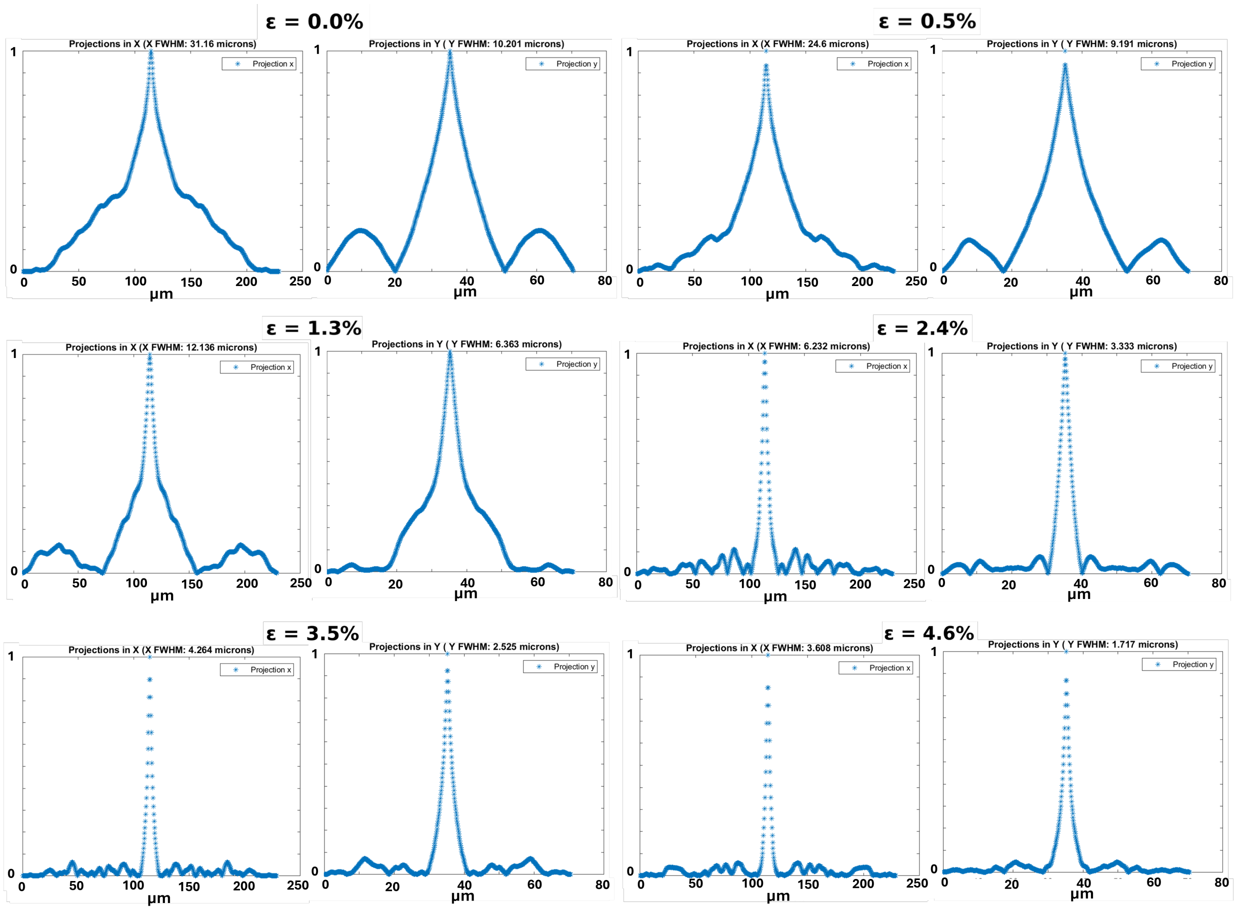

Figure S26. Auto-correlation maps. Center lines of the 2D autocorrelation function for the central layer along directions x_l and y_l.
