# Supplementary material for: Observing formation and evolution of dislocation cells during plastic deformation
Source: Sci Rep. 2025 Mar 13;15:8655. doi: 10.1038/s41598-025-88262-3 (PMC11906882; doi:10.1038/s41598-025-88262-3)
Supplement: Supplementary file 8 — Supplementary Material 8. [file 41598_2025_88262_MOESM8_ESM.pdf]

Supplementary information

Supplementary Information Supplementary Notes I including Figs. S1–S20.

Supplementary Video 1: Center-of-Mass (ϕ,χ) orientation maps. Left: the full field-of-view for the central layer with a region-of-interest, ROI, marked by the rectangular box. Right: a zoom-in corresponding to the ROI. The color scale employed varies from strain level to strain level and is indicated by inverse pole figures inserted.

Supplementary Video 2: Peak broadening maps. The voxel-by-voxel width (FWHM) of the avearge peak broadening $\Delta q = \sqrt{\Delta q_\phi^2 + \Delta q_\chi^2}$, with Δq_ϕ and Δq_χ being the FWHM's resulting from a 2D Gaussian fit to the (ϕ,χ)-distribution. Left: the full field-of-view for the central layer with a region-of-interest, ROI, marked by the rectangular box. Right: a zoom-in corresponding to the ROI.

Supplementary Video 3: Center-of-Mass (ϕ,χ) orientation maps with KAM mask overlaid. The images are replica of those in Supplementary Video 1 with a Kernal-Averaged-Misorientation mask (black lines) overlaid.

Supplementary Video 4: Cell maps. Cells (colored regions) are identified as connected regions using a Kernel-Averaged-Misorientation (KAM) mask (black lines). The colors of the cells represent their average orientation, as indicated by the inverse pole figures inserted. Left: the full field-of-view for the central layer with a region-of-interest, ROI, marked by the rectangular box. Right: a zoom-in corresponding to the ROI.

Supplementary Video 5: Peak broadening maps with KAM masks overlaid. The images are replica of those in Supplementary Video 2 with the KAM mask (black lines) overlaid.
